# O-GlcNAcylation Inhibits Endocytosis of Amyloid Precursor Protein by Decreasing Its Localization in Lipid Raft Microdomains

**DOI:** 10.3390/membranes11120909

**Published:** 2021-11-23

**Authors:** Oh-Hoon Kwon, Yoon Young Cho, Jung Hee Lee, Sungkwon Chung

**Affiliations:** 1 Department of Physiology, Sungkyunkwan University School of Medicine, Suwon 16419, Korea; drummer0114@naver.com (O.-H.K.); myjubilate@skku.edu (Y.Y.C.); 2 Department of Radiology, Samsung Medical Center, Sungkyunkwan University School of Medicine, Seoul 06351, Korea; hijunghee@skku.edu

**Keywords:** Alzheimer’s disease, amyloid precursor protein, O-GlcNAcylation, insulin, lipid raft, endocytosis

## Abstract

Like protein phosphorylation, O-GlcNAcylation is a common post-translational protein modification. We already reported that O-GlcNAcylation of amyloid precursor protein (APP) in response to insulin signaling reduces neurotoxic amyloid-β (Aβ) production via inhibition of APP endocytosis. Internalized APP is delivered to endosomes and lysosomes where Aβ is produced. However, the molecular mechanism involved in the effect of APP O-GlcNAcylation on APP trafficking remains unknown. To investigate the relationship between APP O-GlcNAcylation and APP endocytosis, we tested the effects of insulin on neuroblastoma SH-SY5Y cells overexpressing APP and BACE1, and cultured rat hippocampal neurons. The present study showed that APP O-GlcNAcylation translocated APP from lipid raft to non-raft microdomains in the plasma membrane by using immunocytochemistry and discontinuous sucrose gradients method. By using the biotinylation method, we also found that APP preferentially underwent endocytosis from lipid rafts and that the amount of internalized APP from lipid rafts was specifically reduced by O-GlcNAcylation. These results indicate that O-GlcNAcylation can regulate lipid raft-dependent APP endocytosis via translocation of APP into non-raft microdomains. Our findings showed a new functional role of O-GlcNAcylation for the regulation of APP trafficking, offering new mechanistic insight for Aβ production.

## 1. Introduction

Alzheimer’s disease (AD) is accompanied by the loss of memory functions and other symptoms such as speech and language problems, loss of motivation, and behavioral disorders [1,2]. The number of AD patients is becoming larger with the aging population [3,4]. The major hallmark of AD pathogenesis is characterized by intraneuronal neurofibrillary tangles composed of hyperphosphorylated tau and extracellular amyloid plaques composed of amyloid-β (Aβ) in the brain. Aβ peptides are products of sequential proteolytic cleavage of amyloid precursor proteins (APP) by β- and γ-secretases. APP is synthesized at the endoplasmic reticulum (ER) and transported to the trans-Golgi network (TGN) and the plasma membrane [5]. APP is cleaved by α-secretase at the cell surface most of the time [6]. Some APPs are internalized to endosomes or lysosomes via endocytosis. β-site amyloid precursor protein cleaving enzyme 1 (BACE1), a key enzyme for generating Aβ, is mostly localized in endosomes, lysosomes, and TGN [7,8,9]. Because APP metabolism is affected by APP trafficking and localization, it is very important to understand the association between APP and trafficking factors [10,11,12].

Many studies have aimed to reverse the development of the AD pathological process by focusing on various targets, including post-translational modifications (PTM) [13,14]. O-GlcNAcylation is a dynamic PTM in which O-linked N-acetylglucosamine (O-GlcNAc) is attached to serine and threonine residues of target proteins [15,16]. Two enzymes control O-GlcNAcylation: O-GlcNAc transferase (OGT) for attaching O-GlcNAc to proteins and O-GlcNAcase (OGA) for removing O-GlcNAc [17]. O-GlcNAcylation might be involved in various biological processes [16]. O-GlcNAcylation is a key component in the regulation of transcription factors [18] and epigenetic programs in the nucleus [19,20,21]. It is also a key regulator of diverse cell signaling pathways such as insulin signaling dynamics [22,23]. It has been reported that levels of O-GlcNAcylated proteins are low in AD patients and down-regulation of O-GlcNAcylation is associated with AD pathology [24,25]. APP undergoes O-GlcNAcylation [26], which increases sAPPα level and reduces Aβ secretion [27]. In addition, we have reported that 1,5-hydroximolactone, an inhibitor of OGA, can decrease APP endocytosis rate [28]. Consequently, APP O-GlcNAcylation increases levels of cell surface APP, resulting in decreased Aβ generation [28]. Moreover, we have recently reported that APP is O-GlcNAcylated in response to insulin, resulting in decreased Aβ production via inhibition of APP internalization [29]. An increasing number of studies have shown that the brain is a target organ for insulin [30,31] and that insulin signaling is dysregulated in AD brains [32,33,34,35,36,37,38,39]. In addition, intranasal administration of insulin can improve cognitive impairment [40]. However, molecular mechanisms involved in the effect of insulin on APP trafficking and Aβ production are currently unknown.

Evidence supports that abnormal condition of cholesterol in the brain is implicated in neurodegenerative diseases [41]. A recent study suggests that levels of cholesterol at the plasma membrane are increased about 10% in AD brains [42]. High cholesterol levels are known to affect APP metabolism to increase Aβ production [43]. Cholesterol-rich microdomains called lipid rafts exist in the plasma membrane. These unique structures have various sizes ranging from 5 to 700 nm [44,45] with distinct physiological functions such as the recruitment of proteins, a platform of cell signaling, an entry site of a pathogen, and endocytosis place [44,45,46,47,48,49]. Many studies have shown a relationship between lipid rafts and amyloidogenic processing. It has been reported that β-secretase, four core subunits of the γ-secretase complex, and full-length APP are associated with lipid raft domains [50,51,52]. In addition, elevated cholesterol levels in the cell surface can promote binding between APP and lipid raft-associated adaptor proteins leading to the formation of endocytosis vesicle [53,54], which may increase Aβ generation [55]. These results show the important roles of lipid rafts in APP trafficking and amyloidogenic processing of APP [56,57]. Recently, we reported that the level of cholesterol regulates APP localization in lipid raft microdomains and affects lipid raft-dependent APP endocytosis [58,59].

In the current study, we showed that O-GlcNAcylated APP in response to insulin could reduce the localization of APP in lipid rafts using neuroblastoma SH-SY5Y cells expressing APP and BACE1 as well as cultured hippocampal neurons from Sprague Dawley rats. This study also showed that lipid raft microdomains were sites where APP would undergo endocytosis and that APP O-GlcNAcylation in response to insulin could inhibit APP endocytosis by translocating APP to non-raft microdomains.

## 2. Materials and Methods

### 2.1. Cell Culture and Experimental Treatments

SH-SY5Y cells transfected with wild type APP (695 isoform) and BACE1 (SH-SY5Y-APP/BACE1, maximum number of passages = 26) were cultured at 37 °C in 5% CO_2_ with Dulbecco’s Modified Eagle’s Medium (DMEM) supplemented with 10% (*v*/*v*) heat-inactivated fetal bovine serum (FBS), 100 units/mL penicillin, and 260 μg/mL Zeocin. Cells were treated with 1 μM insulin (Human Recombinant Zinc, Gibco, #12585014) for 2 h. Cells were incubated with 10 μM Akti (Akt inhibitor, Calbiochem, #124018) or 50 μM OSMI-1 (OGT inhibitor, Sigma-Aldrich, #SML1621) for 2 h. Cells were incubated with 50 μM PUGNAc (OGA inhibitor, Tocris Bioscience, Bristol, UK, #3384) or 10 μM Thiamet G (OGA inhibitor, Sigma, Burlington, MA, USA, #SML0244) for 24 h. SH-SY5Y-APP/BACE1 cells were provided by T.-W. Kim (Columbia University Medical Center) [60].

### 2.2. Rat Primary Hippocampal Neuron Culture

Embryonic 18-day-old Sprague-Dawley fetuses (RRID: RGD_734476) from 11 weeks of age female rats were used for hippocampal neuron culture. Rats were purchased from Samtako Bio Korea. The starting breeders of the following strain have been supplied from Taconic Farms, Inc. to Samtako Bio Korea. All procedures were carried out in accordance with the guidelines of the Sungkyunkwan University Animal Care and Ethics Committee. A total of 4 female rats were used in this study, and at least 8 embryonic fetuses were used per pregnant rat for each experiment. We used isoflurane for anesthesia. After receiving animals from the vendor, they were kept in cages for 1~2 h before sacrifice. The hippocampus from both hemispheres was dissected and rinsed with Hank’s balanced salt solution (HBSS). Then, hippocampi were incubated with HBSS containing 0.25% trypsin-EDTA at 37 °C for 5 min. Next, the tissue was incubated with 4 mL HBSS containing 1 mL FBS at 37 °C for 3 min to inactivate trypsin-EDTA, and then the tissue was washed three times with HBSS. The tissue was resuspended in neurobasal medium and filtered through a 100 μm strainer to dissect tissue into single neurons. Then, neurons were washed with HBSS and resuspended in neurobasal medium supplemented with 2% B27, 2 mM glutamax, and 1% penicillin/streptomycin (all supplements from GIBCO). Finally, neurons were grown on poly-D-lysine coated glass coverslips and were maintained for 21 to 23 days (DIV21-DIV23).

### 2.3. Colocalization of APP and Lipid Rafts

SH-SY5Y-APP/BACE1 cells were grown on poly-D-lysine coated cover glass. After washing with ice-cold phosphate-buffered saline (PBS), cells were incubated with APP antibody (1:100, 6E10, BioLegend, monoclonal, RRID: AB_2564652) at 4 °C for 1 h to label cell surface APP. Then, cells were fixed with 4% paraformaldehyde containing 4% sucrose for 15 min and permeabilized in PBS containing 0.1% Triton X-100/2% bovine serum albumin (BSA) for 5 min. After washing, cells were blocked with PBS containing 1% BSA for 1 h, and then cells were incubated with caveolin antibody (1:100, cav-1; BD Transduction Laboratories, polyclonal, RRID: AB_397471) for 2 h in blocking buffer to detect lipid rafts. Following washing with PBS, cells were incubated with goat anti-mouse conjugated with Alexa Fluor 488 (1:200, Invitrogen, RRID: AB_2534069) and donkey anti-rabbit conjugated with Alexa Fluor 647 (1:200, Invitrogen, RRID: AB_2536183) secondary antibodies in blocking buffer at 4 °C for 16 h to label primary antibodies of APP and caveolin, respectively. The next day, cells were washed with PBS and mounted with mounting medium (Sigma, #F6182). Immunofluorescence staining was monitored on a confocal microscope (LSM710, Zeiss).

For colocalization with cholera toxin B (CTB), SH-SY5Y-APP/BACE1 cells were incubated with 6E10 and 10 μg/mL FITC-conjugated CTB (Sigma, #C1655) at 4 °C for 1 h before fixation. Then, cells were processed as described above.

Primary hippocampal neurons were grown on poly-D-lysine coverslips and stained with 6E10 to label endogenous APP at the plasma membrane and 7 μg/mL FITC-conjugated CTB to label lipid rafts at 4 °C for 1 h. After fixing with 4% paraformaldehyde containing 4% sucrose for 15 min, cells were permeabilized in PBS containing 0.1% Triton X-100/2% BSA for 5 min. After 1 h blocking in PBS containing 1% BSA, neurons were incubated with α-NeuN antibody (1:100, Millipore, polyclonal, RRID: AB_10807945) for 2 h at room temperature to detect neurons. Following washes with PBS, cells were incubated with goat anti-mouse conjugated with Alexa Fluor 568 (1:100, Invitrogen, RRID: AB_2534072) and donkey anti-rabbit conjugated with Alexa Fluor 647 (1:100, Invitrogen, RRID: AB_2536183) in blocking buffer overnight to detect primary antibodies of 6E10 and α-NeuN, respectively. The next day, cells were washed and mounted with mounting medium. Immunofluorescence reactivity was captured on a confocal microscope (LSM710, Zeiss). The threshold was automatically set as the 60~100% value. JACoP (Just Another Co-localization Plug-in) of ImageJ program approach (https://imagej.net/Colocalization_Analysis) was used for colocalization of APP with caveolin, APP with CTB, and APP with EEA1. The noise signal intensity variation was eliminated by the subtract background tool in imageJ with the same rolling ball radius value. Mander’s M1 and M2 overlap coefficient was used for the estimation of colocalization [61].

### 2.4. Lipid Raft Fractionation

SH-SY5Y-APP/BACE1 cells were washed twice with ice-cold PBS and harvested with 0.25% trypsin-EDTA. Cells were then lysed in detergent-free condition using morpholinoethanesulfonic acid-buffered saline (MBS; 25 mM MES, 150 mM NaCl, pH 6.5) containing 500 mM sodium carbonate (Sigma, #S7795) and a protease inhibitor cocktail (Millipore, #535140). Cell lysates were homogenized 20 times with a 2 mL homogenizer and 10 times through a 22-gauge needle. Next, cell lysates were sonicated for 1 min (20 s sonication followed by 10 s intervals). Equal amounts of proteins were added to 0.8 mL of 80% (*w*/*v*) sucrose in MBS. Then, 1.6 mL of 35% (*w*/*v*) sucrose and 5% (*w*/*v*) sucrose in MBS were placed in a 5.1 mL ultracentrifuge tube (Beckman Coulter, #326819) to form a discontinuous sucrose gradient. These tubes were placed in a Beckman SW 55Ti rotor (Beckman Coulter) and centrifuged at 50,000 rpm for 3 h at 4 °C. From the top to the bottom, 12 fractions (0.4 mL each) were collected.

### 2.5. Transferrin Uptake

Cells were incubated 25 μg/mL Alexa Fluor 488-conjugated transferrin (Invitrogen, #T-13342) in PBS containing 0.1% BSA at 37 °C for 5 min to allow internalization. After washing surface transferrin with pH 5.5 buffer (0.1 M sodium acetate, 0.05 M NaCl) for 5 min, cells were lysed in MBS buffer containing 500 mM sodium carbonate. Lipid raft and non-raft fractions were isolated as described above. Lipid raft (#4–6) and non-raft fractions (#8–12) were separately combined, and equal amounts of proteins from raft and non-raft fractions were loaded for Western blotting. Proteins were transferred to a nitrocellulose membrane, and the intensity of the bands was detected using LAS 3000 (Fuji Film) and analyzed by Multi Gauge V3.0.

### 2.6. Immunoprecipitation

To measure levels of O-GlcNAcylated APP in lipid raft (#4–6) and non-raft (#8–12) fractions, protein concentrations of each fraction were measured (Bio-Rad, Tokyo, Japan, #5000006). Equal amounts of proteins (400 μg) from lipid raft and non-lipid raft fractions were incubated with 4 μL of O-GlcNAc antibody (BioLegend, monoclonal, RRID: AB_2629520) or APP antibody (6E10, BioLegend, monoclonal, RRID: AB_2564652) for 2 h at room temperature, and then incubated with Protein G agarose Fast Flow (Millipore, #16266) for 16 h at 4 °C. Immunoprecipitated samples were then washed three times with PBS. Equal volumes of each fraction were loaded on a Western blot to detect O-GlcNAcylated APP.

### 2.7. Western Blotting

The proteins were loaded on 10% Tris-glycine SDS-PAGE gel and transferred to 0.2 μm nitrocellulose membrane. The transferred membrane was blocked with 5% (*w*/*v*) non-fat dried milk in Tris-buffered saline with 1% Tween-20 (TBST) for 1 h at room temperature. After washing blocked membrane with PBS four times for 10 min each, the membrane was incubated with the following primary antibodies: APP (1:2000, 6E10, BioLegend, monoclonal, RRID: AB_2564652; 1:2000, 22C11, Invitrogen, monoclonal, RRID: AB_2572978), caveolin (1:10,000, BD Transduction Laboratories, polyclonal, RRID: AB_397471), BACE1 (1:2000, abcam, RRID: AB_10861218), nicastrin (1:1000, CHEMICON, RRID: AB_2235791) and β-actin (1:10,000, EnoGene, monoclonal, #E12-041) at 4 °C for 16 h. Next, the membrane was washed with TBST four times and incubated with horseradish peroxidase-conjugated goat anti-rabbit IgG (1:10,000, Invitrogen, polyclonal, RRID: AB_2533967) or goat anti-mouse IgG (1:2000, Invitrogen, polyclonal, RRID: AB_2536527) antibodies for 1 h at room temperature to detect each primary antibody. After incubation with secondary antibodies, the membrane was washed again with TBST four times. We used an enhanced chemiluminescence reagent (Westsave, #LF-QC0101), and signals were captured with film (MTC Bio, #A8815). The intensity of bands was measured by the LAS-3000 system (Fuji Film) and analyzed by Multi Gauge V3.0.

### 2.8. The Localization of Surface APP in Lipid Raft Microdomains

To monitor the localization of cell surface APP in lipid raft and non-lipid raft microdomains, SH-SY5Y-APP/BACE1 cells were treated with PBS supplemented with 0.25 mg/mL sulfo-NHS-biotin (Thermo, #21217) at 4 °C for 10 min to label all cell surface proteins. The remaining biotins were washed out with 100 mM glycine in PBS three times. After cells were collected, a discontinuous sucrose gradient was performed as described above. Equal amounts of proteins from lipid raft and non-lipid raft fractions were incubated with streptavidin-agarose slurry (Millipore, #16–126) at 4 °C for 16 h to pull down biotin-labeled proteins. After washing, the biotin-labeled proteins were analyzed by Western blot to detect APP, β-actin, and lipid raft marker caveolin.

### 2.9. Rate of Endocytosis of Surface APP in Lipid Raft Microdomains

SH-SY5Y-APP/BACE1 cells were washed with ice-cold PBS to block protein trafficking at the cell surface. All procedures were performed on ice. Cells were incubated with PBS containing 0.25 mg/mL NHS-SS-biotin (Thermo, #21441) at 4 °C for 10 min to label all surface proteins. Excess biotin was washed out with 100 mM glycine in PBS three times, and cells were incubated with 1% BSA in PBS for 15 min at 4 °C. After washing, cells were incubated at 37 °C for 5 min to allow internalization of biotin-labeled surface proteins. Then, cells were quickly placed on ice and washed with ice-cold PBS to stop internalization. The remaining cell surface-bound biotin was removed by incubating cells with reducing agent (50 mM sodium-2-mercapoethanesulfomate, 150 mM NaCl, 1 mM EDTA, 0.2% BSA, 20 mM Tris HCl, pH 8.6) twice for 25 min each at 4 °C. Then, the reducing agent was quenched by ice-cold 5 mg/mL iodoacetamide in 1% BSA for 10 min. After cells were collected, a discontinuous sucrose gradient was performed as described above to obtain lipid raft and non-lipid raft fractions. Biotin-labeled internalized proteins were pulled down from both fractions as described above. APP, β-actin, and caveolin were monitored by Western blot.

### 2.10. Colocalization of APP with Early Endosomes

SH-SY5Y-APP/BACE1 cells were grown on poly-D lysine coated glass coverslips. Surface APP was labeled with 6E10 antibody at 4 °C for 1 h, and then, cells were transferred to 37 °C in order to allow internalization. Internalization was stopped with ice-cold PBS and cells were fixed with 4% paraformaldehyde containing 4% sucrose at room temperature for 15 min. Next, cells were permeabilized with PBS containing 0.1% Triton X-100/2% BSA for 5 min. Then, cells were blocked with 2% BSA in PBS for 1 h. Cells were incubated with EEA1 (1:100, Cell Signaling, monoclonal, RRID: AB_2096811) antibody to detect early endosome in blocking buffer for 2 h at room temperature. Following washing with PBS, cells were incubated with goat anti-mouse conjugated with Alexa Fluor 647 (1:100, Invitrogen, RRID: AB_2535809) and goat anti-rabbit conjugated with Alexa Fluor 488 (1:100, Invitrogen, RRID: AB_2576217) secondary antibodies in blocking buffer to detect APP and EEA1, respectively. The next day, cells were washed with PBS and mounted with mounting medium. Immunofluorescence staining was monitored on a confocal microscope (LSM710, Zeiss, Jena, Germany). Colocalization of APP and early endosomes was calculated as described above for APP and lipid rafts.

### 2.11. Statistical Analysis

No randomization was performed to allocate subjects in this study. Also, no blinding was performed. No sample calculation was performed. This study was exploratory. No exclusion criteria were pre-determined. The data were analyzed using OriginPro (OriginLab, Seoul, Korea). All statistical analyses were conducted using one-way ANOVA between controls and drug-treated groups. Statistical significance was considered when *p*-value was less than 0.05. Data are expressed as the mean ± SEM.

## 3. Results

### 3.1. Insulin Decreases APP Localization in Lipid Rafts

**Figure 1:**
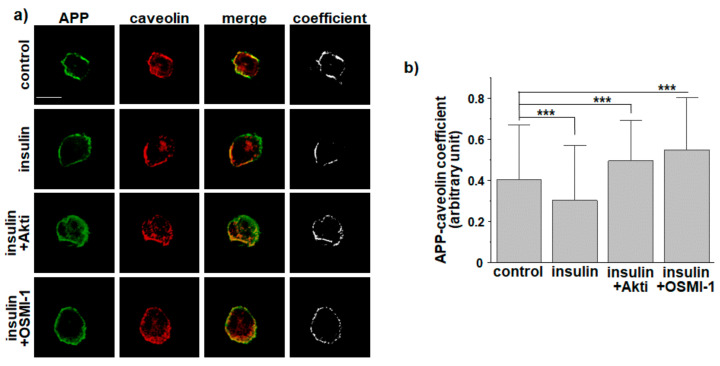
Insulin reduces APP localization in lipid raft microdomains. SH-SY5Y-APP/BACE1 cells were incubated with 1 μM insulin for 2 h. Akt inhibitor (Akti, 10 μM) or OGT inhibitor (OSMI-1, 50 μM) was also used. After washing, cells were incubated with 6E10 antibody at 4 °C to label APP at the plasma membrane. Cells were then fixed, permeabilized, and incubated with caveolin antibody to label lipid rafts. Fluorescent conjugated secondary antibodies were used. (**a**) Typical immunoreactivities of APP (green) and caveolin (red) are shown. (**b**) Coefficients of APP and caveolin were quantified as arbitrary units using Zen software (*n* = 40, number of cells were randomly selected from four independent experiments). The threshold was automatically set as the 60100% value. (Mander’s M1 coefficient: control, 0.4025 ± 0.01967; insulin, 0.30248 ± 0.01868; Akti, 0.49505 ± 0.01759; OSMI-1, 0.55038 ± 0.02195; fraction of A overlapping B, image A: APP, image B: caveolin). Scale bar is 10 μm. One-way ANOVA: ***, *p* < 0.001. All values represent the mean ± SEM.

We have previously reported that insulin decreases Aβ production by regulating APP processing via APP O-GlcNAcylation [29]. We also showed that insulin increases cell surface APP by decreasing APP endocytosis rate. Lipid rafts play a role in membrane trafficking and endocytosis of various proteins [46]. In addition, many studies have indicated that amyloidogenic APP processing occurs mainly in lipid raft microdomains [50,51,52,55,56,57]. From these results, we hypothesized that insulin affects lipid raft-dependent APP endocytosis via APP O-GlcNAcylation. To test this hypothesis, we measured the localization of cell surface APP in lipid raft by co-staining APP with caveolin, a marker for lipid rafts. SH-SY5Y-APP/BACE1 cells were incubated with 1 μM insulin for 2 h and then treated with APP antibody at 4 °C to label APP at the plasma membrane. After fixation and permeabilization, cells were incubated with caveolin-1 antibody. Typical immunoreactivities of APP and caveolin are shown in Figure 1a. Microscopic images with more cells are shown in Figure S1. Colocalization of caveolin and APP were quantified and are shown in Figure 1b. The coefficient was significantly decreased by 22.4% (*n* = 40, number of cells) in insulin-treated cells compared to control cells, indicating that insulin decreased APP localization in lipid rafts. As another marker for lipid rafts, we used cholera toxin B (CTB), which binds to a component of lipid rafts called ganglioside GM1 [62]. Insulin significantly decreased the colocalization of APP and CTB (Figure S2), which was consistent with the result in Figure 1.

Effects of insulin on APP O-GlcNAcylation and APP endocytosis are mediated by Akt signaling and OGT [29]. Thus, we tested the effect of insulin on APP localization in lipid rafts in the presence of Akt inhibitor (Akti) or OGT inhibitor (OSMI-1). Inhibitory effects of insulin on colocalization of APP and caveolin were completely blocked in the presence of Akti or OSMI-1, indicating that the insulin effect was mediated by Akt and OGT signaling (Figure 1a,b). It was noteworthy that levels of colocalization were higher in the presence of these inhibitors than in control cells, indicating basal activities of Akt or OGT in control conditions as we observed previously [29].

**Figure 2:**
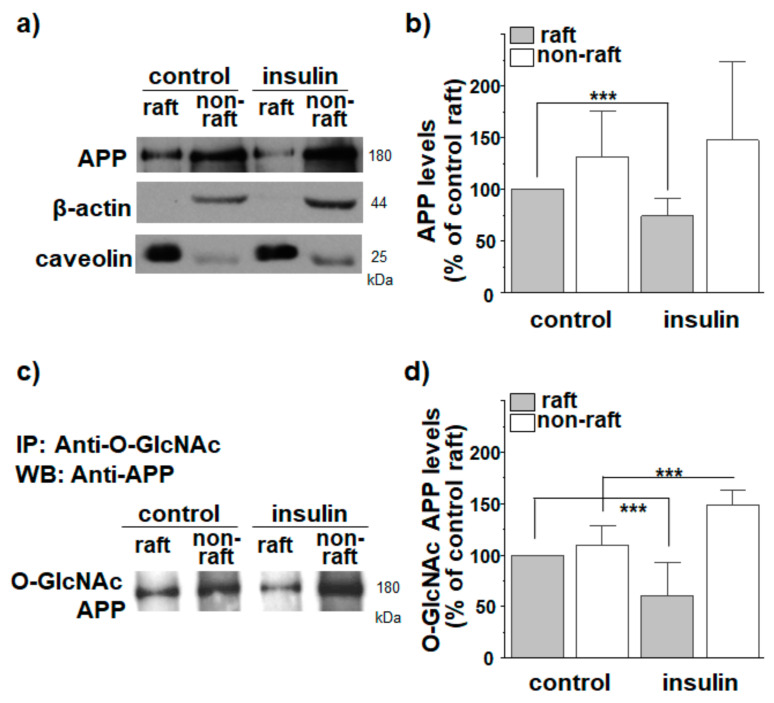
O-GlcNAcylation in response to insulin translocates APP from lipid raft to non-raft fractions. SH-SY5Y-APP/BACE1 cells were incubated with insulin for 2 h. Cells were then harvested, homogenized, and sonicated. Equal amounts of proteins from cell lysates were loaded on discontinuous sucrose density gradients as described in the Methods section to obtain 12 fractions. We combined fractions 4 to 6 (lipid raft fractions) or fractions 8 to 12 (non-raft fractions). (**a**,**b**) Equal amounts of proteins from lipid raft and non-raft fractions were loaded for Western blots to detect APP (*n* = 7, number of independent experiments). APP levels were compared to APP level in raft fraction of control cells. (**c**,**d**) Lipid raft and non-raft fractions were immunoprecipitated with O-GlcNAc antibody, and then probed with APP antibody (*n* = 7, number of independent experiments). O-GlcNAc APP levels were compared to O-GlcNAc APP levels in raft fractions of control cells. Caveolin was used as a lipid raft marker. One-way ANOVA: ***, *p* < 0.001. All values represent mean ± SEM.

The immunocytochemical method we used to study the localization of APP in lipid rafts may generate ambiguous results due to the insufficient resolution for the small size of lipid raft microdomains. To confirm the colocalization results by using the biochemical method, lipid raft fractions were isolated in detergent-free conditions as described previously [58]. For this purpose, the same amount of proteins from cell lysates were loaded on discontinuous sucrose gradients. After obtaining 12 fractions, equal volumes of each fraction were loaded for Western blot. A typical result for APP distribution is shown in Figure S3. The majority of APP was localized in non-raft fractions. However, a small but significant amount of APP was localized in lipid raft fractions, which was consistent with the previous report [58]. When levels of cholesterol and protein from each fraction were measured, cholesterol-high lipid raft fractions (fractions 4 to 6) were clearly separated from protein-high non-raft fractions (fractions 8 to 12). Moreover, caveolin was enriched in fractions 4 to 6. Protein levels in each fraction did not differ between control and insulin-treated cells. Total APP level was not changed by insulin, as observed previously [29]. For a better quantification of APP levels, we separately combined lipid raft and non-raft fractions. Equal amounts of proteins from both raft and non-raft fractions were loaded for Western blotting. A typical result is shown in Figure 2a. APP levels were then compared to the APP levels from lipid raft fractions in control cells (Figure 2b; *n* = 7, number of independent experiments). Thus, we compared the relative APP levels out of total proteins from each fraction. Although the majority of APP was localized in non-raft fractions (Figure S3), the combined APP level in lipid raft fractions was considerably high. This was because APP comprised a larger proportion out of total proteins in lipid raft fractions. Insulin significantly decreased APP levels in raft fractions by 26%. In contrast, insulin increased APP levels in non-raft fractions although it was not statistically significant. These results are consistent with decreased APP localization in lipid rafts by insulin.

Transferrin receptor is commonly used as a non-raft marker [63,64,65]. Since transferrin is internalized through transferrin receptor via clathrin-dependent endocytosis [66], transferrin is also used as a non-raft marker [67]. The level of total transferrin was not changed by insulin (Figure S4). After allowing internalization, we measured transferrin levels from lipid raft and non-raft fractions. Transferrin fluorescence was detected only in non-raft fractions, confirming that we could isolate raft fractions free from non-raft fractions in detergent-free experimental condition (Figure S4). Importantly, insulin did not affect clathrin-dependent endocytosis, which was consistent with the previous reports [29]. The level of caveolin was not changed by insulin treatment (Figure S5).

### 3.2. APP O-GlcNAcylation in Response to Insulin Translocates APP from Lipid Rafts to Non-Rafts

We measured levels of O-GlcNAcylated APP in raft and non-raft fractions using the immunoprecipitation method. First, O-GlcNAcylated proteins were pulled down from raft and non-raft fractions using an antibody against O-GlcNAc. APP antibody was then used to detect O-GlcNAcylated APP. A typical result is shown in Figure 2c. Relative O-GlcNAcylated APP levels in each fraction were calculated as shown in Figure 2d (*n* = 7, number of independent experiments). In control cells, O-GlcNAcylated APP showed similar distributions between raft and non-raft fractions. Insulin significantly decreased O-GlcNAcylated APP level in raft fractions by 39%. Conversely, the O-GlcNAcylated APP level in non-raft fractions was increased from 110% to 149% in response to insulin. To confirm this result, we first pulled down APP from raft and non-raft fractions using APP antibody and then detected O-GlcNAcylated APP using O-GlcNAc antibody. Insulin decreased O-GlcNAcylated APP level in lipid raft fractions, which was consistent with results in Figure 2c,d (Figure S6). Insulin increased O-GlcNAcylated APP level in non-rafts, although it was not statistically significant. Thus, these results may suggest that O-GlcNAcylation translocates APP from lipid rafts into non-raft microdomains.

**Figure 3:**
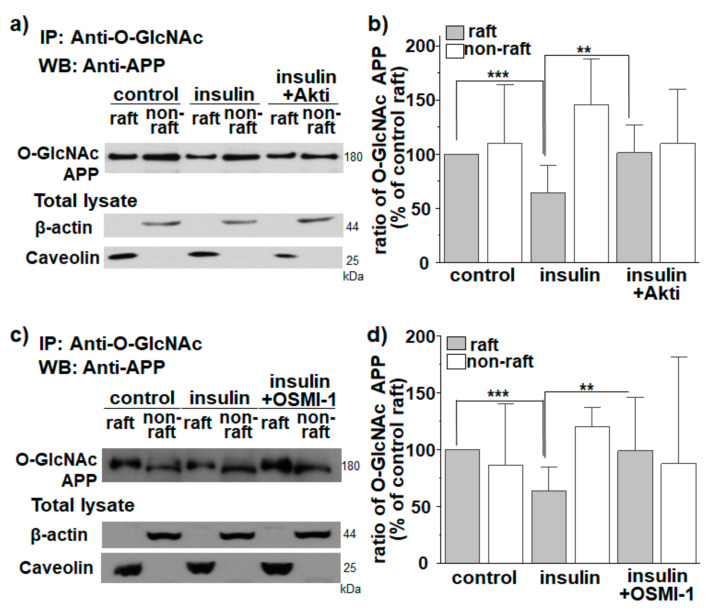
Effect of insulin on APP localization in lipid rafts occurs via Akt signaling and OGT. SH-SY5Y-APP/BACE1 cells were incubated with 1 μM insulin for 2 h. Akt inhibitor (Akti, 10 μM) or OGT inhibitor (OSMI-1, 50 μM) was also used. O-GlcNAc APP levels were compared to O-GlcNAc APP levels in raft fraction of control cells. (**a**,**b**) The effect of insulin on the localization of O-GlcNAcylated APP in lipid rafts in the presence of Akti (*n* = 7, number of independent experiments). (**c**,**d**) The effect of insulin on localization of O-GlcNAcylated APP in lipid rafts in the presence of OSMI-1 (*n* = 7, number of independent experiments). Caveolin was used as a lipid raft marker. One-way ANOVA: **, *p* < 0.01; ***, *p* < 0.001. All values represent the mean ± SEM.

Then, we tested whether the effect of insulin on translocation of O-GlcNAcylated APP into non-raft fractions occurred via Akt signaling and OGT. In the presence of Akt inhibitor, the effect of insulin on localization of O-GlcNAcylated APP in lipid rafts was completely prevented (Figure 3a,b; *n* = 7, number of independent experiments). A similar result was obtained with an OGT inhibitor (Figure 3c,d; *n* = 7, number of independent experiments). These results suggest that the effect of insulin on translocation of O-GlcNAcylated APP into non-rafts occurred via Akt signaling and OGT. The level of caveolin was not changed either by Akt inhibitor or OGT inhibitor (Figure S5).

To ascertain that the reduced APP localization in lipid rafts was mediated by O-GlcNAcylation, we used OGA inhibitors, PUGNAc and Thiamet G [28]. Both PUGNAc (Figure S7) and Thiamet G (Figure S8) decreased APP localization in lipid rafts without affecting APP levels, supporting that insulin effect on APP localization in lipid rafts was mediated by APP O-GlcNAcylation.

APP is one of hundreds of proteins modified by O-GlcNAcylation [26]. O-GlcNAcylated proteins can be found in cytoplasmic, nuclear, mitochondrial, and plasma membrane compartments. It is known that BACE1 and two components of γ-secretase, Aph-1 and nicastrin, are targeted to lipid rafts by post-translational palmitoylation of C-terminal cysteine residues near their transmembrane domains [68,69]. Moreover, both BACE1 and nicastrin undergo O-GlcNAcylation [70,71]. Considering these results, we tested whether O-GlcNAcylation in response to insulin regulated the localization of BACE1 and nicastrin in lipid rafts. Insulin increased the level of O-GlcNAcylated BACE1 and decreased the localization of O-GlcNAcylated BACE1 in lipid raft fractions (Figure S9). Similar results were obtained with nicastrin (Figure S10). Thus, O-GlcNAcylation in response to insulin reduced the localization of both BACE1 and nicastrin in lipid raft microdomains.

Next, we investigated whether insulin could regulate the localization of total O-GlcNAcylated proteins in lipid rafts using an antibody against O-GlcNAc. A typical Western blot result is shown in Figure S11. Insulin treatment increased levels of O-GlcNAcylated proteins. Also, insulin increased levels of O-GlcNAcylated proteins both in lipid raft and non-raft fractions as shown previously [29]. However, insulin did not change the distribution of total O-GlcNAcylated proteins in lipid raft and non-raft fractions. Thus, insulin-mediated O-GlcNAcylation affected the localization of some proteins, such as APP, BACE1, and nicastrin, but not O-GlcNAcylated proteins in general.

### 3.3. APP O-GlcNAcylation in Response to Insulin Decreases APP Internalization from Lipid Rafts

**Figure 4:**
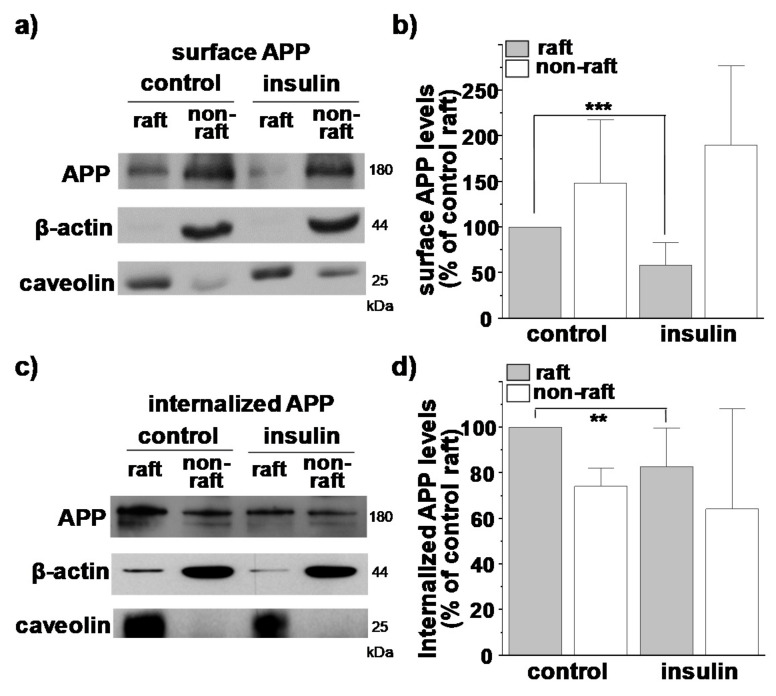
O-GlcNAcylation decreases APP internalization from lipid rafts. SH-SY5Y-APP/BACE cells were incubated with insulin for 2 h. (**a**) To examine APP localization at the plasma membrane, cells were incubated with EZ-Link NHS-biotin for 10 min at 4 °C to label all surface proteins. Biotin-labeled cell lysates were then loaded onto discontinuous sucrose density gradient to separate lipid raft and non-lipid raft fractions as described in the Methods section. A typical result shows surface APP levels in lipid raft and non-raft fractions. (**b**) Quantitative analysis of surface APP was shown (*n* = 7, number of independent experiments). Surface APP levels were compared to the levels of surface APP in raft fractions of control cells. (**c**) To examine whether APP endocytosis occurred in lipid rafts, cells were incubated with EZ-Link NHS-SS-biotin for 10 min at 4 °C to label surface proteins. Cells were then incubated at 37 °C for 5 min to allow internalization of biotin-labeled surface proteins. After internalization, remaining surface-bound biotin was removed, and biotin-labeled cell lysates were loaded onto a discontinuous sucrose density gradient to separate lipid raft and non-lipid raft fractions as described in the Methods section. Internalized biotin-bound APP levels in lipid raft and non-raft fractions were determined with Western blots. (**d**) Analysis of band densitometry showing levels of internalized APP (*n* = 7, number of independent experiments). Levels of internalized APP were compared to the APP levels internalized from lipid rafts in control condition. Caveolin was used as a lipid raft marker. One-way ANOVA: **, *p* < 0.01; ***, *p* < 0.001. All values represent the mean ± SEM.

Previously, we have shown that O-GlcNAcylation decreases the rate of APP endocytosis [29], and cholesterol level affects lipid raft-dependent APP internalization [59]. However, whether O-GlcNAcylation in response to insulin affects APP internalization remains unclear. Although we obtained APP levels in raft and non-raft fractions using discontinuous sucrose gradients (Figure 2a), APP might exist in intracellular organelles in addition to the cell surface. For this reason, we quantitatively measured APP levels localized only in cell surface using biotin-labeled lipid raft fractionation. Cells were incubated with EZ-Link sulfo-NHS-biotin at 4 °C for 10 min to label all cell surface proteins. The remaining unbound biotins were then washed out. The same amounts of proteins from biotin-bound cell lysates were loaded onto a discontinuous sucrose density gradient. After 12 fractions were obtained, we separately combined lipid raft (fractions 4 to 6) and non-raft fractions (fractions 8 to 12). Equal amounts of biotin-bound proteins from both raft and non-raft fractions were then pulled down with streptavidin beads. Thus, bead-captured APP represented cell surface APP localized either in lipid raft or non-raft fractions. A typical result is shown in Figure 4a. Surface APP levels were compared to the levels of surface APP from lipid rafts in control cells (Figure 4b). Insulin decreased the cell surface APP level in raft fractions by 42% (*n* = 7, number of independent experiments). Reciprocally, the cell surface APP level in non-raft fractions was increased by insulin although it was not significant. Thus, O-GlcNAcylation translocated cell surface APP from lipid rafts into non-rafts, which was consistent with the results shown in Figure 2a.

Next, we combined the reversible biotinylation method with lipid raft fractionation to specifically measure the internalized APP from lipid rafts and non-rafts in the plasma membrane. Cells were incubated with EZ-Link NHS-SS-biotin to label all surface proteins, followed by incubating cells at 37 °C for 5 min to allow internalization of biotin-labeled surface proteins. All remaining surface-bound biotins were then removed with a reducing agent. Lipid raft and non-raft fractions were isolated and pulled down with streptavidin beads from equal amounts of proteins. Then, equal volumes of each fraction were loaded for Western blotting as described above. We assumed that characteristics of internalized membranes from lipid raft and non-raft fractions were maintained as in the plasma membrane. Thus, bead-captured APP represented internalized APP originated only from cell surface during 5 min of incubation time. A typical result is shown in Figure 4c. Internalized APP levels were compared to levels of internalized APP from lipid rafts in control cells (Figure 4d). In control cells, the amount of internalized APP originated from lipid rafts was significantly larger than that from non-rafts, although the majority of surface APP was localized in non-raft fractions (Figure 4b). This result strongly indicated that APP preferentially internalized from lipid rafts as we have reported previously [59]. More importantly, insulin decreased the amount of internalized APP from lipid rafts (Figure 4d), which was consistent with decreased APP levels in lipid raft fractions by insulin (Figure 2a). If endocytosis of APP occurred indiscriminately from lipid rafts and from non-rafts, the amount of internalized APP originated from non-raft fractions should have increased by insulin since insulin increased APP levels in non-raft fractions (Figure 4b). Insulin, however, did not change the amount of internalized APP originated from non-raft fractions. Together, these results indicated that APP is preferentially internalized from lipid rafts, and that O-GlcNAcylation of APP in response to insulin decreased APP endocytosis from lipid rafts by translocating APP into non-raft microdomains.

To confirm that the internalized APP observed in Figure 4c was originated from the plasma membrane, cells were incubated with EZ-Link NHS-SS-biotin to label cell surface proteins at 4 °C for 10 min, followed by treating with a reducing agent to remove all surface biotin. Then, cells were incubated at 37 °C for 5 min to allow endocytosis. Total APP level was decreased in lipid raft fractions by insulin, which was consistent with our previous results (Figure S12). The internalized APP was not detected both in raft and non-raft fractions when we used streptavidin bead to pull down the biotin-labeled proteins. Since we removed biotin from the plasma membrane, the absence of APP indicated that the internalized APP observed in Figure 4c was originated from the plasma membrane.

### 3.4. O-GlcNAcylation in Response to Insulin Promotes Translocation of Endogenous APP into Non-Rafts from Cultured Hippocampal Neurons

**Figure 5:**
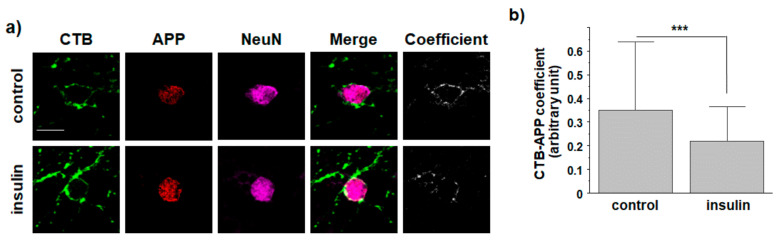
Insulin reduces the localization of endogenous APP in lipid raft microdomains from rat primary hippocampal neurons. Cultured hippocampal neurons were treated with 1 μM insulin for 2 h in neurobasal media, and then incubated with cholera toxin B (CTB) and 6E10 antibody at 4 °C to label lipid rafts and APP at the plasma membrane, respectively. After washing, cells were fixed and incubated with NeuN antibody for 2 h to identify neurons. (**a**) Typical immunoreactivities of CTB and APP are shown. (**b**) Intensities were quantified as arbitrary units with an LSM710 microscope (Zeiss) using Zen software (*n* = 30, number of cells were randomly selected from four different experiments). The threshold was automatically set as the 60–100% value. (Mander’s M2 coefficient: control, 0.4548 ± 0.01484; insulin, 0.33535 ± 0.01475; fraction of B overlapping A, image A: CTB, image B: APP). Scale bar is 10 μm. One-way ANOVA: ***, *p* < 0.001. All values represent the mean ± SEM.

We next tested whether insulin affected the localization of APP in lipid rafts from primary hippocampal neurons from Sprague-Dawley rats. To monitor the localization of APP at the plasma membrane, cells were co-stained with APP antibody and CTB. Neurons were incubated with 1 μM insulin for 2 h. After cells were fixed and permeabilized, they were incubated with NeuN for 2 h to identify neurons. Typical immunoreactivities of APP and CTB are shown in Figure 5a. Intensities were quantified and shown in Figure 5b (*n* = 30, number of cells from four independent experiments). The coefficient of APP and CTB was significantly decreased by 39.6% in insulin-treated neurons compared to that in control neurons. This result suggested that insulin could promote the translocation of endogenous APP from lipid raft to non-raft microdomains in neurons. Since we have already confirmed the reduction in Aβ42 secretion by insulin from cultured cortical neurons in a previous study [29], our results suggest that insulin might affect APP endocytosis and Aβ production in neurons.

## 4. Discussion

**Figure 6:**
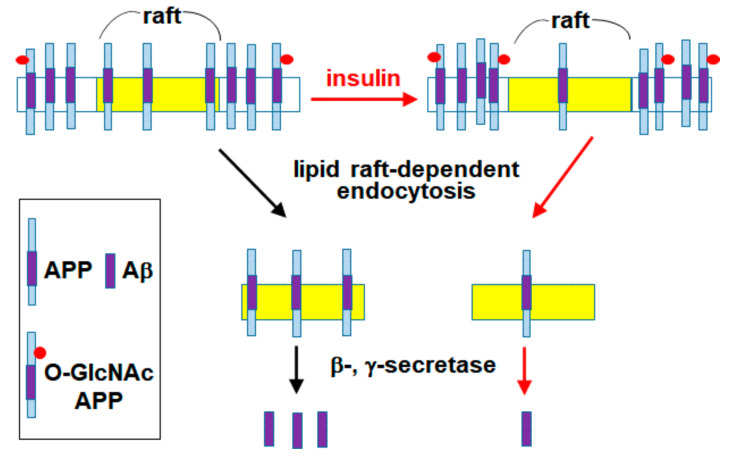
Our current model. APP O-GlcNAcylation in response to insulin signaling translocated APP from lipid rafts into non-rafts. Since APP would preferentially undergo endocytosis from lipid rafts and be cleaved by β-, γ-secretases in early endosomes and lysosomes, APP O-GlcNAcylation inhibited Aβ production.

We have previously reported that APP O-GlcNAcylation in response to insulin signaling can promote the non-amyloidogenic pathway by decreasing APP endocytosis [29]. The present study showed that O-GlcNAcylated APP in response to insulin decreased APP localization in lipid rafts and consequently decreased lipid raft-dependent APP endocytosis. A schematic model summarizing our results is shown in Figure 6. We have recently shown that a significant amount of APP is localized in lipid rafts, and that up-regulation of cellular cholesterol levels can increase APP localization in lipid rafts [58]. Also, we reported that cholesterol levels affect the rate of lipid raft-dependent APP internalization [59]. It is well known that APP metabolism is affected by APP trafficking and APP localization in intracellular compartments [10,11,12]. Thus, endocytosis of APP is a crucial step in Aβ generation. APP is synthesized in the ER and transported to TGN and the plasma membrane where it is predominantly cleaved by α-secretase [6]. Alternatively, APP in the plasma membrane is internalized via endocytosis. APP is then sorted into endosomes or lysosomes where it is likely to be cleaved by β-secretase [7,8,9].

Since we confirmed that insulin decreased lipid raft-dependent APP endocytosis, we determined the subcellular localization of internalized APP from the plasma membrane. For this purpose, surface APP was labeled with APP antibody, followed by incubating at 37 °C for 5 min to allow APP endocytosis. Cells were then fixed, permeabilized, and incubated with EEA1 antibody (early endosomes marker) to measure the colocalization of APP and EEA1. At 5 min, a large amount of APP was localized at early endosomes in control cells (Figure S13). In insulin-treated cells, the coefficient of APP and EEA1 was reduced by about 34%, indicating that insulin reduced the amount of APP reaching endosomes by inhibiting APP internalization from the plasma membrane. Since endosomes are the site for Aβ production, the reduced APP reaching endosomes might be the reason for the deceased Aβ generation by insulin [29].

Lipid raft microdomains play an important role in the trafficking and internalization of proteins [54,72]. Therefore, the distribution of membrane proteins between lipid rafts and non-rafts might be critical for lipid raft-dependent endocytosis. Many studies support that lipid rafts are closely associated with the amyloidogenic pathway of APP [46,50,51,52,55,56]. In this study, we showed that APP O-GlcNAcylation in response to insulin promoted the translocation of APP into non-raft fractions, resulting in the reduction in lipid raft-dependent APP internalization. It is known that lipid raft microdomains are dynamic membranes, which are greatly affected by environmental factors such as cholesterol level, protein content, lateral pressure, and temperature [73]. Therefore, obtaining lipid raft microdomains is a very difficult task, even though improved biophysical and biochemical tools are available [74,75]. To separate lipid rafts from non-rafts, we used the biochemical method in non-detergent conditions as in previous experiments [58,59]. Despite the clear separation of raft from non-raft fractions in these experiments, the distribution of proteins between rafts and non-rafts showed significant variation in some experiments. In addition, we compared the relative levels of APP or O-GlcNAcylated APP out of total proteins. Although the majority of APP was localized in non-raft fractions (Figure S3), APP comprised a larger proportion out of total proteins in lipid raft fractions compared to non-raft fractions. The larger amount of APP out of total proteins in rafts might give a significant insulin effect on APP translocation. Conversely, the degree of changes in APP translocation by insulin might be smaller in non-raft fractions. This may explain why most of our data for insulin effects reached significant levels in rafts rather than in non-rafts.

It is possible that APP can be internalized by various pathways including clathrin-dependent and -independent endocytosis. In this study, we found that transferrin was detected only in non-raft fractions, and the level of internalized transferrin from the plasma membrane was not altered by insulin treatment. We also found that APP is internalized both from non-rafts and from lipid rafts, although lipid rafts are preferential sites for APP internalization. Taken together, our results may suggest a specific regulation of APP endocytosis from lipid rafts by O-GlcNAcylation in response to insulin.

During transit in the secretory pathway, APP is subjected to a variety of post-translational modifications, including proteolytic processing, glycosylation, ubiquitination, sulfation, palmitoylation, and phosphorylation. The primary function of palmitoylation is to enhance hydrophobicity, resulting in lipid rafts localization of the protein [76]. It has been reported that APP undergoes palmitoylation. Palmitoylated APP is not only targeted at lipid rafts, but it is also a preferential substrate for β-secretase, and consequently Aβ production [77]. Thus, the effect of APP O-GlcNAcylation was opposite to that of APP palmitoylation in terms of APP localization in lipid rafts and Aβ production. It is possible that the addition of a single O-GlcNAc to APP might decrease hydrophobicity, resulting in decreased localization in lipid rafts. It is also noteworthy that the lipid rafts localization of BACE1 and nicastrin, important proteins for the amyloidogenic pathway of APP, are also regulated by insulin via O-GlcNAcylation.

Several studies have already reported that the intracellular YENPTY motif in the C-terminal residue of APP is involved in its endocytosis [10]. This motif interacts with many endocytic adaptor proteins such as Fe65, Mint, and Dab1, resulting in regulation of cell surface APP levels and Aβ production [10,78,79]. It is also known that a large fraction of APP forms dimers to promote Aβ generation [80,81,82]. In addition, palmitoylation of APP affects its dimerization and localization in lipid rafts [83]. Considering these results, APP O-GlcNAcylation may affect the interaction with endocytic adaptor proteins or affect APP oligomerization, resulting in localization changes in lipid raft microdomains as well as APP internalization. However, we could not exclude the possibility that the effects of insulin on APP endocytosis may occur via the changes in O-GlcNAcylation of other proteins, such as endocytic adaptors regulating APP internalization. Since insulin-like growth factor 1 (IGF-1) is similar in molecular structure to insulin, both insulin and IGF-1 can bind to each other’s receptors and mediate similar responses. It is reported that IGF-1 influences the level of O-GlcNAcylation [84,85]. Considering these possibilities, further studies would be needed to test the effects of IGF-1 on APP O-GlcNAcylation and APP endocytosis.

In summary, O-GlcNAcylation modification of APP translocates APP from lipid raft to non-raft microdomains in the plasma membrane. Since APP preferentially undergoes endocytosis from lipid rafts, the amount of internalized APP from lipid rafts is specifically reduced by O-GlcNAcylation. Thus, O-GlcNAcylation regulates lipid raft-dependent APP endocytosis via translocation of APP from lipid rafts to non-raft microdomains. Our findings show a novel function of O-GlcNAcylation for the regulation of APP trafficking, which could offer a new therapeutic opportunity to reduce Aβ production.

## Supplementary Materials

The following are available online at https://www.mdpi.com/article/10.3390/membranes11120909/s1, Figure S1. Insulin reduces APP localization in lipid raft microdomains. Figure S2. Insulin decreases the colocalization coefficients of APP and cholera toxin B. Figure S3. The distribution of APP, proteins, and cholesterol levels in 12 fractions obtained from discontinuous sucrose gradients. Figure S4. The effect of insulin on transferrin internalization. Figure S5. The expression levels of caveolin were not changed by insulin, Akti, and OSMI-1. Figure S6. The effects of insulin on the translocation of O-GlcNAcylated APP into non-raft fractions by using immunoprecipitation with APP antibody. Figure S7. The effects of PUGNAc on the translocation of O-GlcNAcylated APP into non-raft fractions. Figure S8. The effects of Thiamet G on the translocation of O-GlcNAcylated APP from lipid raft to non-raft fractions. Figure S9. Insulin induced translocation of O-GlcNAcylated BACE1 from lipid raft to non-raft fractions. Figure S10. Insulin induced translocation of O-GlcNAcylated nicastrin from lipid raft to non-raft fractions. Figure S11. Effect of insulin on O-GlcNAcylated protein levels in lipid raft and non-raft fractions. Figure S12. Internalized biotin-labeled proteins were originated from plasma membrane. Figure S13. Insulin decreased the localization of internalized APP in early endosomes.
