# Supplementary material for: O-GlcNAcylation Inhibits Endocytosis of Amyloid Precursor Protein by Decreasing Its Localization in Lipid Raft Microdomains
Source: Membranes (Basel). 2021 Nov 23;11(12):909. doi: 10.3390/membranes11120909 (PMC8704492; doi:10.3390/membranes11120909)
Supplement: Supplementary file 1 [file membranes-11-00909-s001.zip › membranes-1447899-supplementary.pdf]

Supplementary materials

O-GlcNAcylation inhibits endocytosis of amyloid precursor protein by decreasing its localization in lipid raft microdomains.

Oh-Hoon Kwon[1,#], Yoon Young Cho[1,#], Jung Hee Lee[2], Sungkwon Chung[1,*]

[1] Department of Physiology, Sungkyunkwan University School of Medicine, Suwon 16419, South Korea

[2] Department of Radiology, Samsung Medical Center, Sungkyunkwan University School of Medicine, Seoul 06351, South Korea

*Correspondence to:
Professor Sungkwon Chung, PhD.
Department of Physiology, Sungkyunkwan University School of Medicine
Suwon 440-746, South Korea
schung@skku.edu
Tel: 82-31-299-6103
Fax: 82-31-299-6129

[#]These authors contributed equally

Figure S1

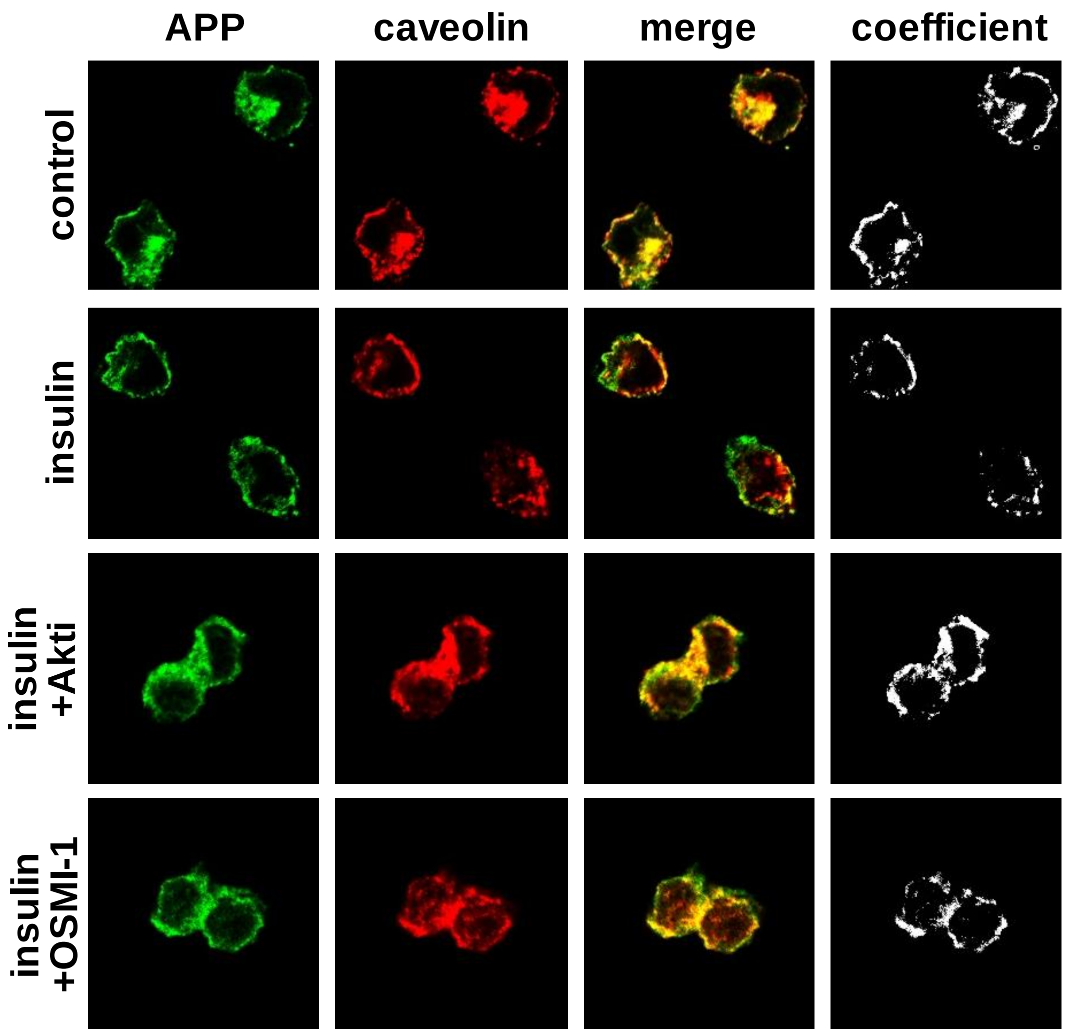

Figure S1. Insulin reduces APP localization in lipid raft microdomains. SH-SY5Y-APP/BACE1 cells were incubated with 1 μM insulin for 2 h. Akt inhibitor (Akti, 10 μM) or OGT inhibitor (OSMI-1, 50 μM) was also used. After washing, cells were incubated with 6E10 antibody at 4℃ to label APP at the plasma membrane. Cells were then fixed, permeabilized, and incubated with caveolin antibody. After labeling fluorescent conjugated secondary antibodies, typical immunoreactivities of APP (green) and caveolin (red) are measured. Scale bar is 10 μm.

Figure S2

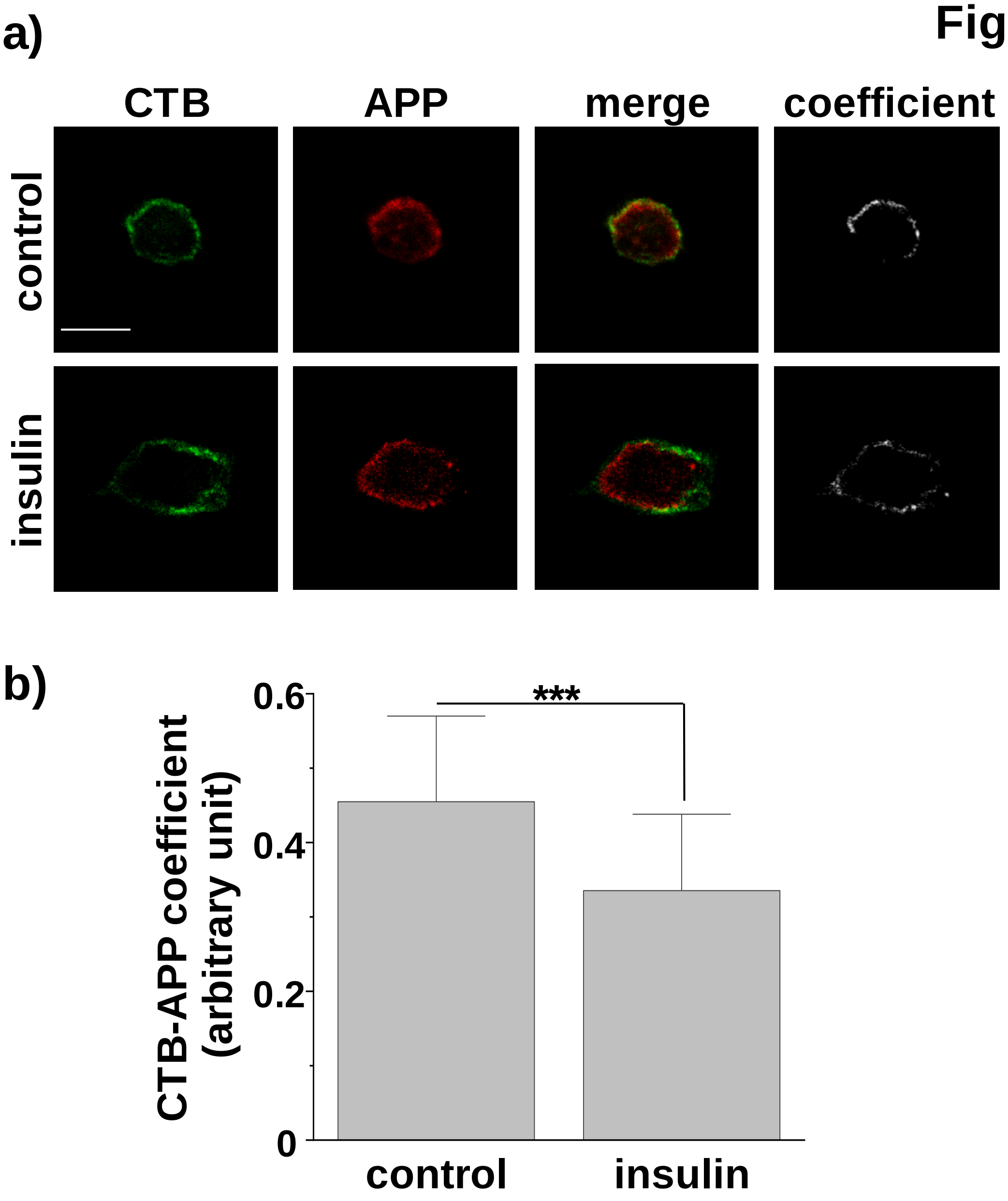

a)
CTB
APP
merge
coefficient
control
insulin
b)
CTB-APP coefficient (arbitrary unit)
0.6
0.4
0.2
0
***
control
insulin

Figure S2. Insulin decreases the colocalization coefficients of APP and cholera toxin B. SH-SY5Y-APP/BACE1 cells were incubated with 1 μM insulin. After washing, cells were incubated with 6E10 antibody and 10 μg/ml FITC-conjugated cholera toxin B subunits (CTB; Sigma, #C1655) at 4°C for 1 h to label APP and lipid raft microdomains, respectively. Cells were then fixed and incubated with fluorescent conjugated secondary antibody. (a) Typical immunoreactivities of CTB (green) and APP (red) are shown. (b) The intensities were quantified as the arbitrary unit by LSM710 microscope (Zeiss) using Zen software (n=20, number of cells. Randomly sectected 5 cells in 4 different experiments.). The threshold was automatically set as the 60-100% value. (Manders' M2 coefficient: control, 0.35007±0.02381; insulin, 0.21993±0.01285; fraction of B overlapping A, image A: CTB, image B: APP). Scale bar is 10 μm. One way ANOVA: ***, $p < 0.001$. All values represent mean ± SEM.

Figure S3

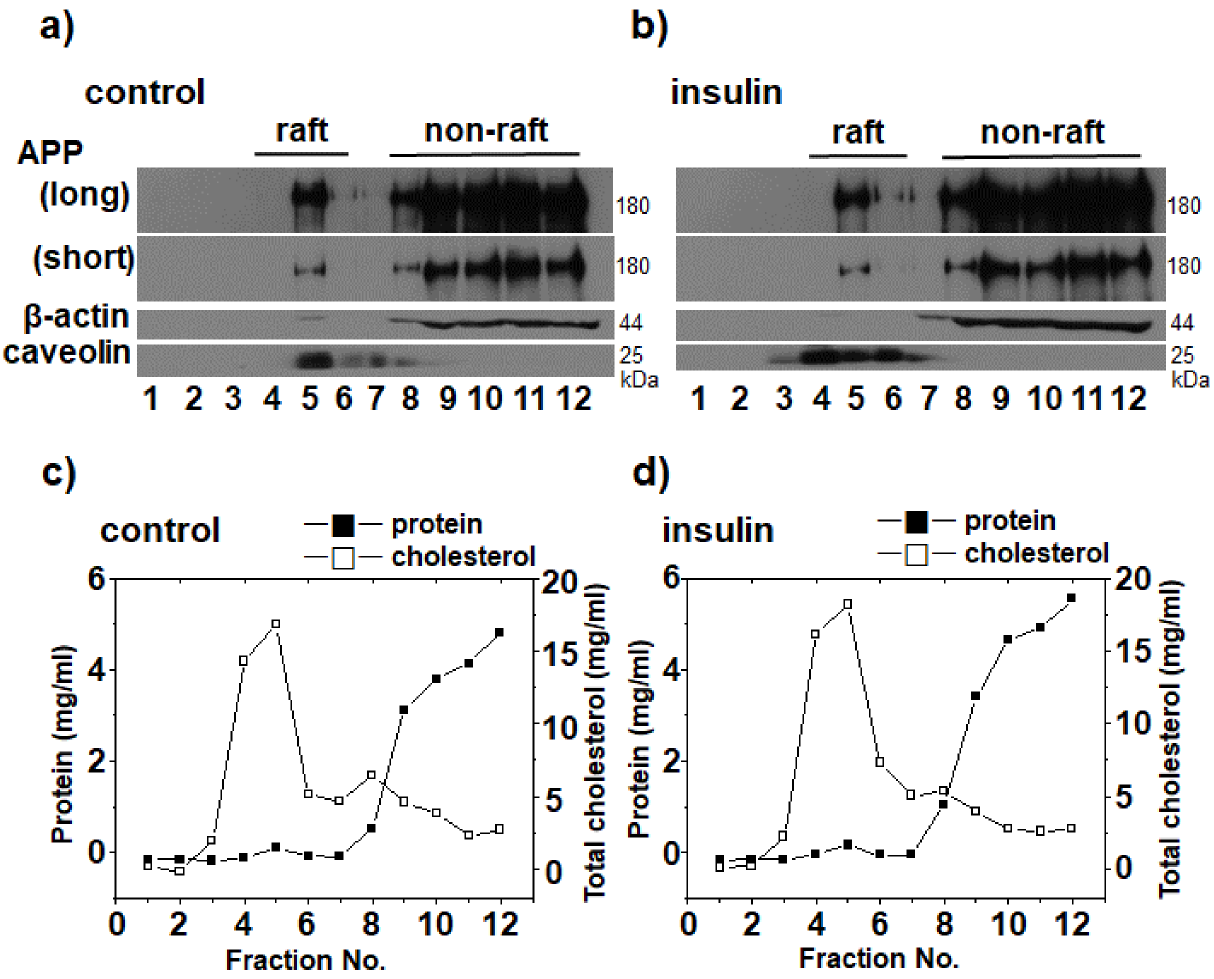

Figure S3. The distribution of APP, proteins, and cholesterol levels in 12 fractions obtained from discontinuous sucrose gradients. SH-SY5Y-APP/BACE1 cells were homogenized with sodium carbonate buffer as described in Methods. The cell lysates were loaded on discontinuous sucrose density gradients. The gradients were harvested from top to bottom to obtain 12 fractions, and equal volumes of each fraction were loaded on SDS gels. (a, b) A representative western blot shows APP, β-actin, and caveolin (lipid raft marker) expression levels. (c, d) The levels of cholesterol and proteins in each fraction were analyzed using Amplex Red cholesterol assay kit and Bradford method, respectively.

Figure S4

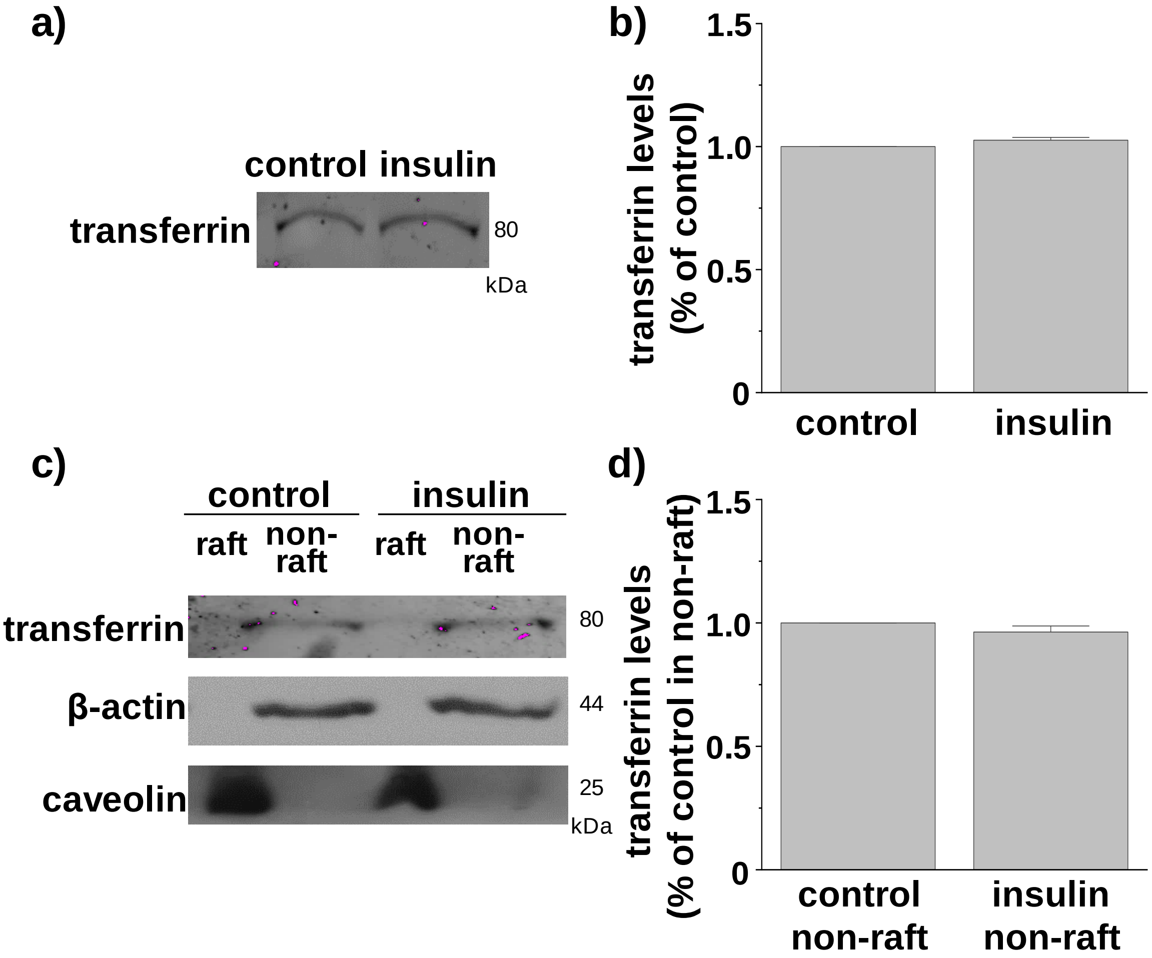
a)
control insulin
transferrin
80
kDa
b)
transferrin levels (% of control)
1.5
1.0
0.5
0
control
insulin
c)
control
insulin
raft
non-raft
raft
non-raft
transferrin
β-actin
caveolin
80
44
25
kDa
d)
transferrin levels (% of control in non-raft)
1.5
1.0
0.5
0
control non-raft
insulin non-raft

Figure S4. The effect of insulin on transferrin internalization. (a, b) SH-SY5Y-APP/BACE1 cells were incubated with 1 μM insulin. Level of transferrin was not changed by insulin when Alexa Fluor 488-transferrin was used (n=3, number of independent cell culture preparations). (c, d) SH-SY5Y-APP/BACE1 cells were incubated with Alexa Fluor 488-transferrin at 37°C for 5 min, following remaining transferrin at the plasma membrane was stripped by acidic buffer. Equal amount of proteins from cell lysates were loaded on discontinuous sucrose density gradients as described in Method section to obtain 12 fractions. We combined fractions 4 to 6 (lipid raft fractions) or fractions 8 to 12 (non-raft fractions). Proteins in the same amount from lipid raft and non-raft fractions were loaded for western blots to detect internalized transferrin. Transferrin fluorescent bands were detected only in non-raft fractions, and the levels of transferrin were not altered by insulin (n=3, number of independent cell culture preparations). A representative western blot shows APP, β-actin, and caveolin (lipid raft marker).

Figure S5

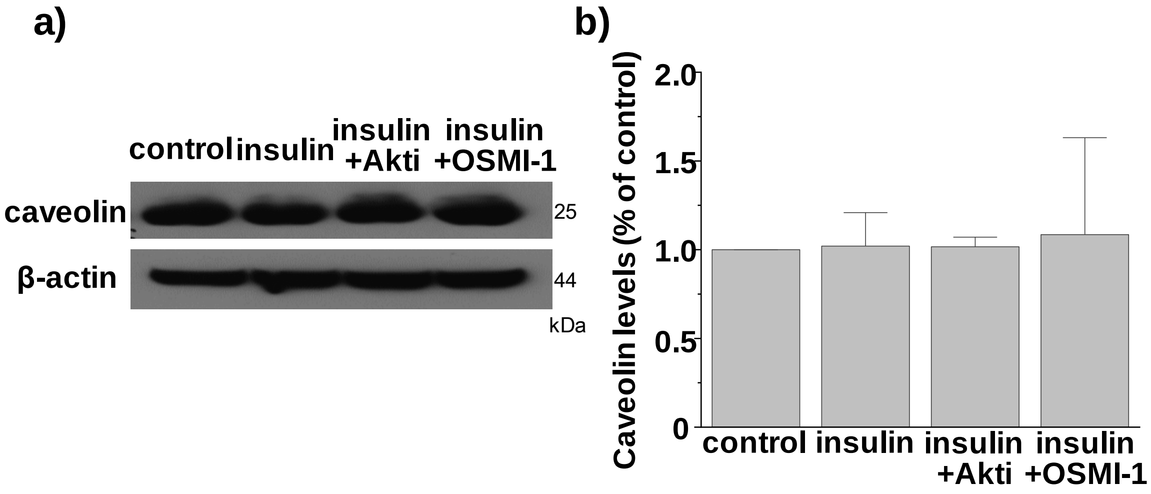

Figure S5. The expression levels of caveolin were not changed by insulin, Akti, and OSMI-1. SH-SY5Y-APP/BACE1 cells were incubated with 1 μM insulin for 2 h. Akt inhibitor (Akti, 10 μM) or OGT inhibitor (OSMI-1, 50 μM) was also used. (a) A representative western blot shows the levels of total caveolin. β-actin was used as a loading control. (b) The levels of total caveolin were not changed in different conditions (n=7, number of independent cell culture preparations).

Figure S6

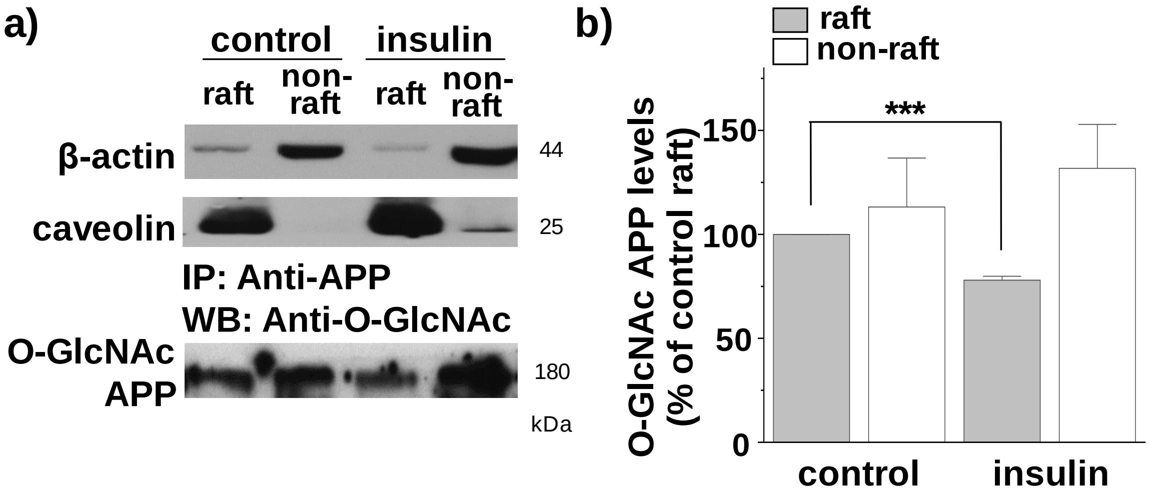

Figure S6. The effects of insulin on the translocation of O-GlcNAcylated APP into non-raft fractions by using immunoprecipitation with APP antibody. (a) SH-SY5Y-APP/BACE1 cells were incubated with 1 μM insulin for 2 h. Cells were then harvested, homogenized, and sonicated. Equal amount of proteins from cell lysates were loaded on discontinuous sucrose density gradients as described in Method section to obtain 12 fractions. We combined fractions 4 to 6 (lipid raft fractions) or fractions 8 to 12 (non-raft fractions). Lipid raft and non-raft fractions were immunoprecipitated with APP antibody and then probed with O-GlcNAc antibody (n=3, number of independent cell culture preparations). Caveolin was used as a lipid raft marker. (b) O-GlcNAc APP levels were compared to O-GlcNAc APP level in raft fraction of control cells. One-way ANOVA: ***, $p < 0.001$. All values represent mean ± SEM.

Figure S7

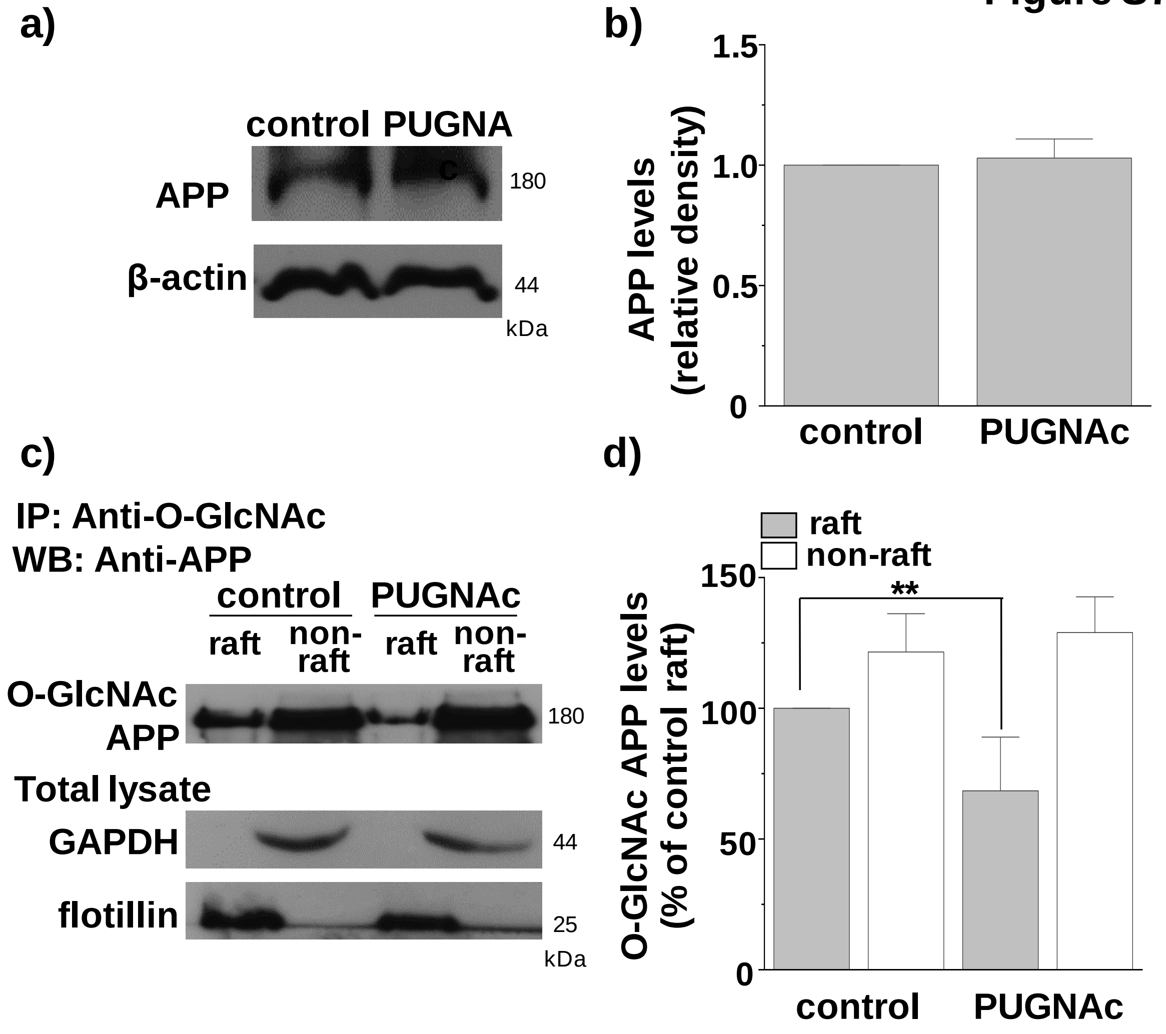
a)
control
PUGNA
c
APP
180
β-actin
44
kDa
b)
1.5
1.0
0.5
0
APP levels
(relative density)
control
PUGNAc
c)
IP: Anti-O-GlcNAc
WB: Anti-APP
control
PUGNAc
raft
non-raft
raft
non-raft
O-GlcNAc APP
180
Total lysate
GAPDH
44
flotillin
25
kDa
d)
raft
non-raft
150
100
50
0
**
O-GlcNAc APP levels
(% of control raft)
control
PUGNAc

Figure S7. The effects of PUGNAc on the translocation of O-GlcNAcylated APP into non-raft fractions. SH-SY5Y-APP/BACE1 cells were incubated with 50 μM PUGNAc for 24 h. (a, b) When cells lysates were loaded for western blotting, APP level was not changed by PUGNAc (n=3, number of independent cell culture preparations). (c, d) Following PUGNAc treatment, cells were harvested, homogenized, and sonicated. Equal amount of proteins from cell lysates were loaded on discontinuous sucrose density gradients as described in Method section to obtain 12 fractions. We combined fractions 4 to 6 (lipid raft fractions) or fractions 8 to 12 (non-raft fractions). Lipid raft and non-raft fractions were immunoprecipitated with O-GlcNAc antibody and then probed with APP antibody (n=3, number of independent cell culture preparations). Caveolin was used as a lipid raft marker. O-GlcNAc APP levels were compared to O-GlcNAc APP levels in raft fraction of control cells. One-way ANOVA: **, $p < 0.01$. All values represent mean ± SEM.

Figure S8

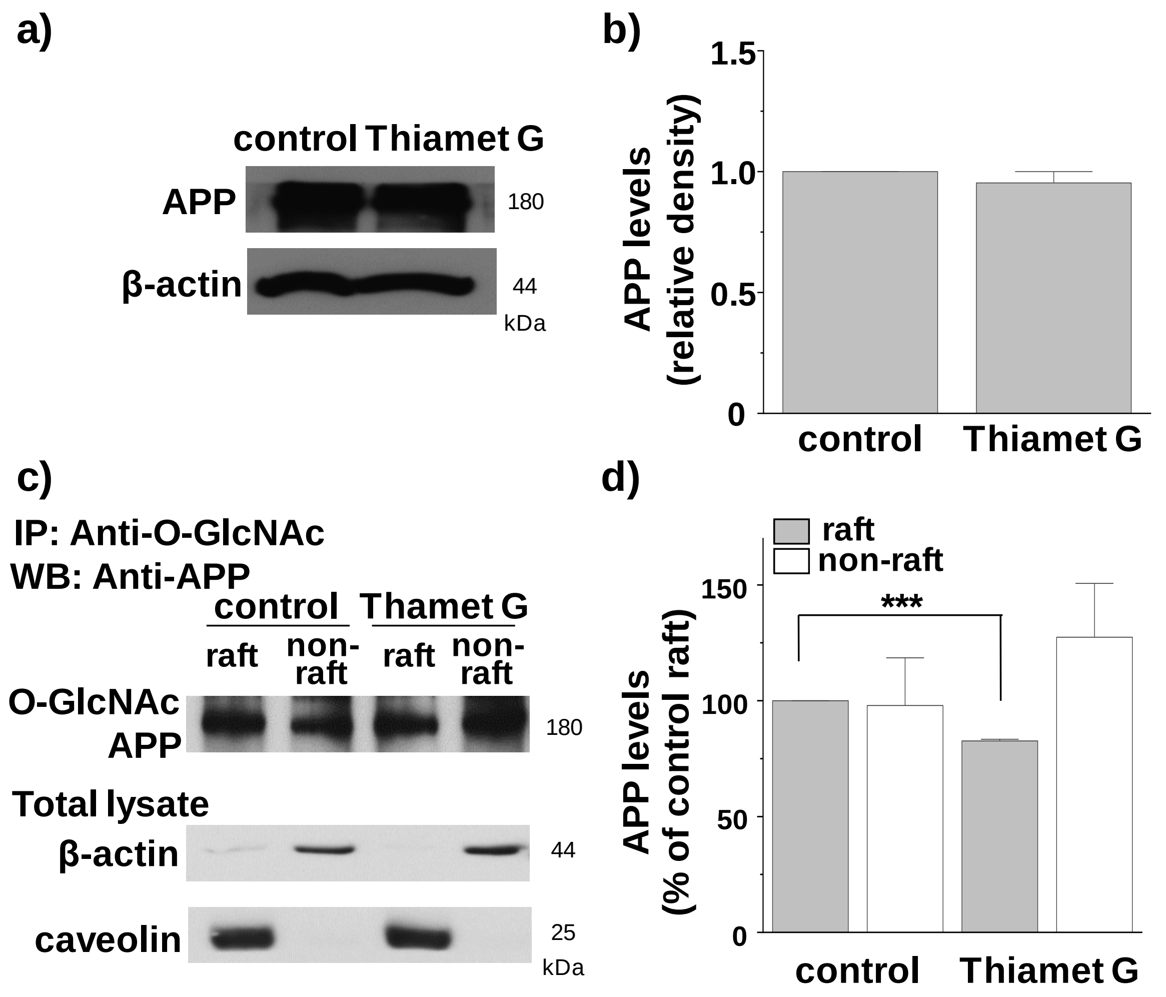
a)
control
Thiamet G
APP
180
β-actin
44
kDa
b)
APP levels
(relative density)
1.5
1.0
0.5
0
control
Thiamet G
c)
IP: Anti-O-GlcNAc
WB: Anti-APP
control
Thamet G
raft
non-raft
raft
non-raft
O-GlcNAc APP
180
Total lysate
β-actin
44
caveolin
25
kDa
d)
raft
non-raft
***
APP levels
(% of control raft)
150
100
50
0
control
Thiamet G

Figure S8. The effects of Thiamet G on the translocation of O-GlcNAcylated APP from lipid raft to non-raft fractions. SH-SY5Y-APP/BACE1 cells were incubated with 10 μM Thiamet G for 24 h. (a, b) When cells lysates were loaded for western blotting, APP level was not changed by Thiamet G (n=3, number of independent cell culture preparations). (c, d) Following Thiamet G treatment, cells were harvested, homogenized, and sonicated. Equal amount of proteins from cell lysates were loaded on discontinuous sucrose density gradients as described in Method section to obtain 12 fractions. We combined fractions 4 to 6 (lipid raft fractions) or fractions 8 to 12 (non-raft fractions). Lipid raft and non-raft fractions were immunoprecipitated with O-GlcNAc antibody and then probed with APP antibody (n=3, number of independent cell culture preparations). Caveolin was used as a lipid raft marker. O-GlcNAc APP levels were compared to O-GlcNAc APP levels in raft fraction of control cells. One-way ANOVA: ***, $p < 0.001$. All values represent mean ± SEM.

Figure S9

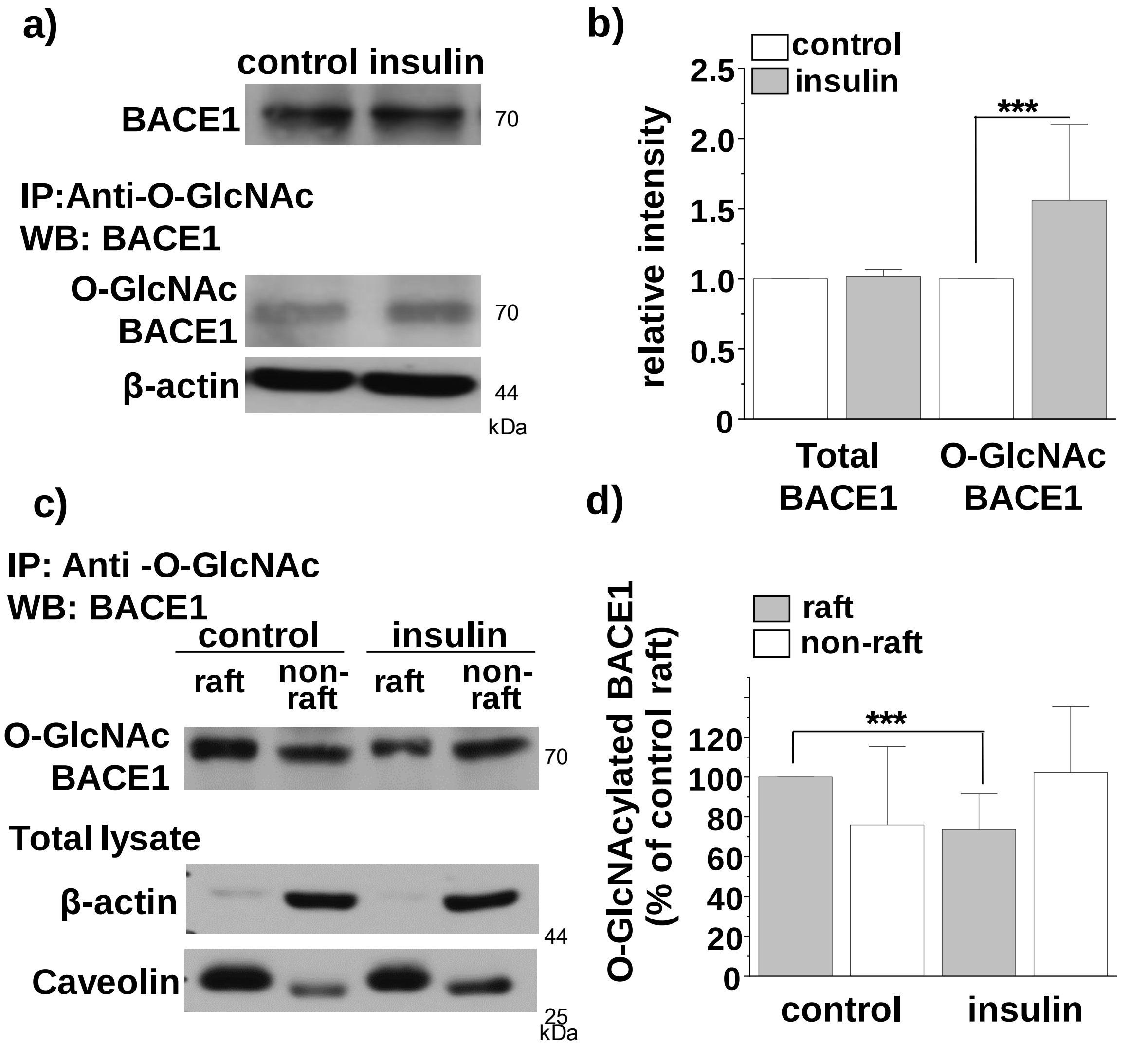

Figure S9. Insulin induced translocation of O-GlcNAcylated BACE1 from lipid raft to non-raft fractions. SH-SY5Y-APP/BACE1 cells were incubated with 1 μM insulin for 2 h, and cell lysates were prepared. (a) A representative western blot shows the levels of total BACE1 and O-GlcNAcylated BACE1. β-actin was used as a loading control. (b) Bars correspond to the densitometric analysis of O-GlcNAcylated BACE1 levels (n=7). (c) SH-SY5Y-APP/BACE1 cells were incubated with 1 μM insulin for 2 h, and then lipid raft and non-raft fractions were obtained. The combined fractions were immunoprecipitated with O-GlcNAc antibody, and then probed with BACE1 antibody. (d) O-GlcNAc BACE1 levels were compared to O-GlcNAc BACE1 level in raft fraction of control cells (n=7). One way ANOVA: ***, $p < 0.001$. All values represent mean ± SEM.

Figure S10

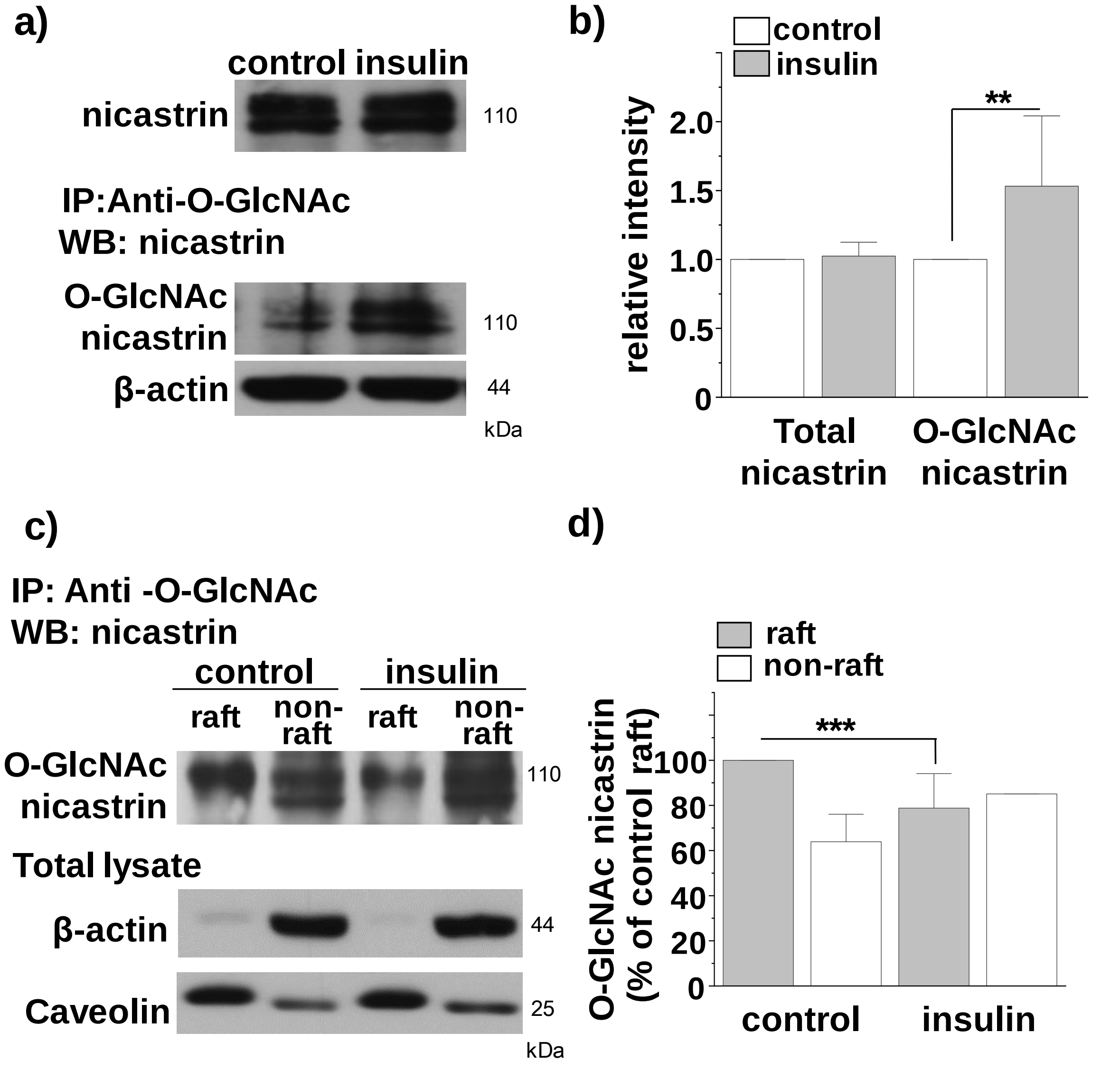
a)
control
insulin
nicastrin
110
IP:Anti-O-GlcNAc
WB: nicastrin
O-GlcNAc
nicastrin
110
β-actin
44
kDa
b)
control
insulin
**
relative intensity
2.0
1.5
1.0
0.5
0
Total
nicastrin
O-GlcNAc
nicastrin
c)
IP: Anti -O-GlcNAc
WB: nicastrin
control
insulin
raft
non-raft
raft
non-raft
O-GlcNAc
nicastrin
110
Total lysate
β-actin
44
Caveolin
25
kDa
d)
raft
non-raft
***
O-GlcNAc nicastrin
(% of control raft)
100
80
60
40
20
0
control
insulin

Figure S10. Insulin induced translocation of O-GlcNAcylated nicastrin from lipid raft to non-raft fractions. SH-SY5Y-APP/BACE1 cells were incubated with 1 μM insulin for 2 h, and cell lysates were prepared. (a) A representative western blot shows the levels of total nicarstrin and O-GlcNAcylated nicastrin. β-actin was used as a loading control. (b) Bars correspond to the densitometric analysis of O-GlcNAcylated nicarstrin levels (n=7). (c) SH-SY5Y-APP/BACE1 cells were incubated with 1 μM insulin for 2 h, and then lipid raft and non-raft fractions were obtained. The combined fractions were immunoprecipitated with O-GlcNAc antibody, and then probed with nicarstrin antibody. (d) O-GlcNAc nicarstrin levels were compared to O-GlcNAc nicarstrin level in raft fraction of control cells (n=7). One way ANOVA: **, $p < 0.01$; ***, $p < 0.001$. All values represent mean ± SEM.

Figure S11

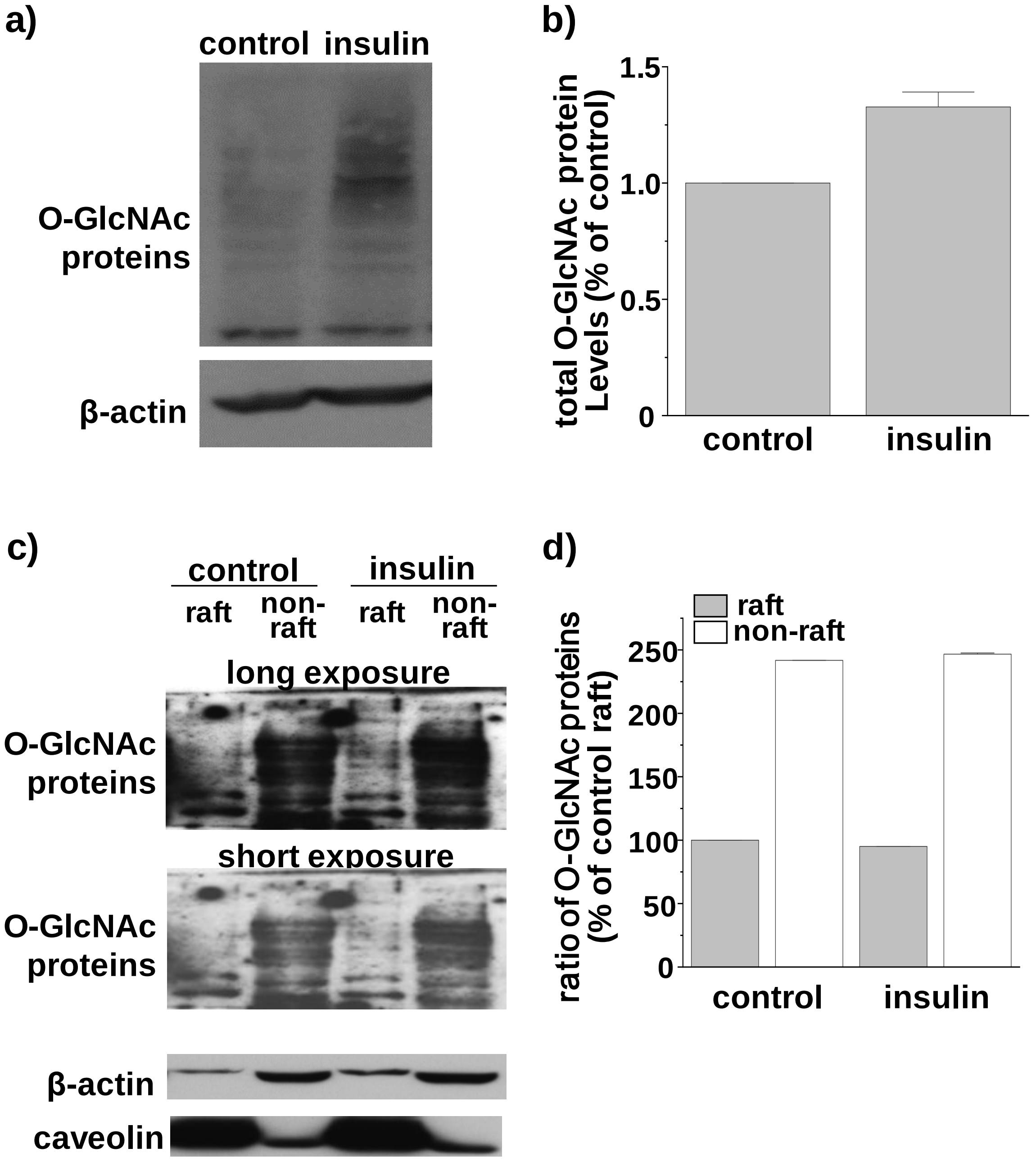

a)
control
insulin
O-GlcNAc proteins
β-actin
b)
total O-GlcNAc protein Levels (% of control)
1.5
1.0
0.5
0
control
insulin
c)
control
insulin
raft
non-raft
raft
non-raft
long exposure
O-GlcNAc proteins
short exposure
O-GlcNAc proteins
β-actin
caveolin
d)
raft
non-raft
ratio of O-GlcNAc proteins (% of control raft)
250
200
150
100
50
0
control
insulin

Figure S11. Effect of insulin on O-GlcNAcylated protein levels in lipid raft and non-raft fractions. SH-SY5Y-APP/BACE1 cells were incubated with insulin for 2 h, and cell lysates were obtained. (a) A representative western blot shows O-GlcNAcylated protein levels from control and insulin-treated cells. (b) Bars correspond to the densitometric analysis of O-GlcNAcylated protein levels (n=4, number of independent cell culture preparations). (c) Cell lysates were obtained and then fractionated into lipid raft and non-raft fractions by using discontinuous sucrose gradient method. A representative western blot shows O-GlcNAcylated protein levels with long- or short-exposure. Caveolin were used as markers for raft fractions. (d) O-GlcNAc protein levels were compared to O-GlcNAc protein level in raft fractions of control cells. (n=4, number of independent cell culture preparations).

Figure S12

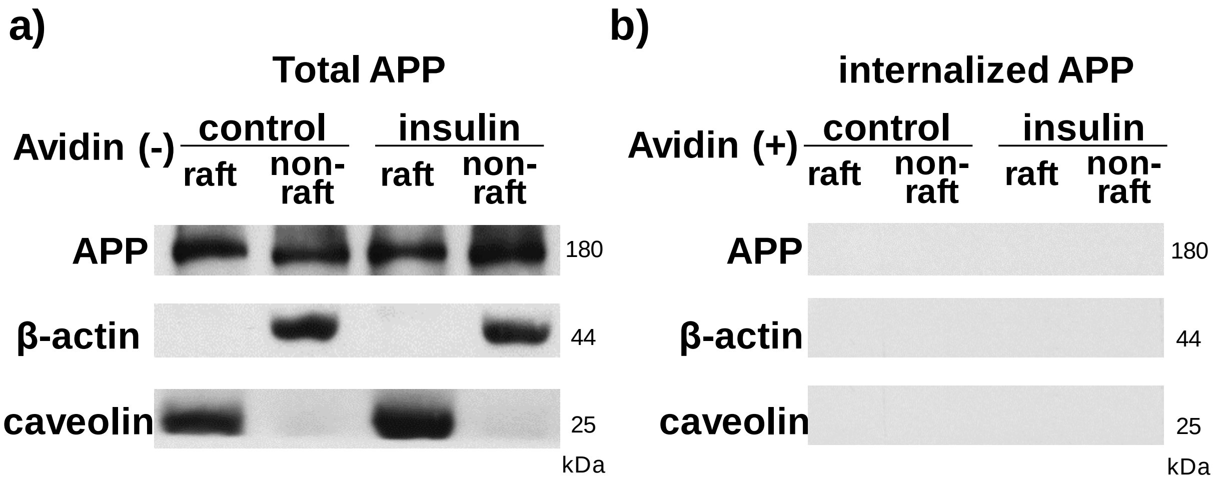

Figure S12. Internalized biotin-labeled proteins were originated from plasma membrane. SH-SY5Y-APP/BACE1 cells were incubated with insulin for 2 h. To confirm whether the internalized APP is originated from plasma membrane, cells were incubated with biotin to label surface proteins for 10 min at 4°C, and then remaining surface biotin was immediately removed with acidic buffer. Cells were then incubated at 37°C for 5 min to allow internalization. After internalization, biotin-labeled cell lysates were loaded onto a discontinuous sucrose density gradient to separate lipid raft and non-lipid raft fractions. We combined fractions 4 to 6 (lipid raft fractions) or fractions 8 to 12 (non-raft fractions). (a) Before pull-down with streptavidin beads, proteins in the same amount from lipid raft and non-raft fractions were loaded for western blots to detect total APP, β-actin, and caveolin. Caveolin was used as a lipid raft marker. (b) After pull-down with streptavidin beads, proteins in the same amount from lipid raft and non-raft fractions were loaded for western blots to detect internalized APP, β-actin, and caveolin.

Figure S13

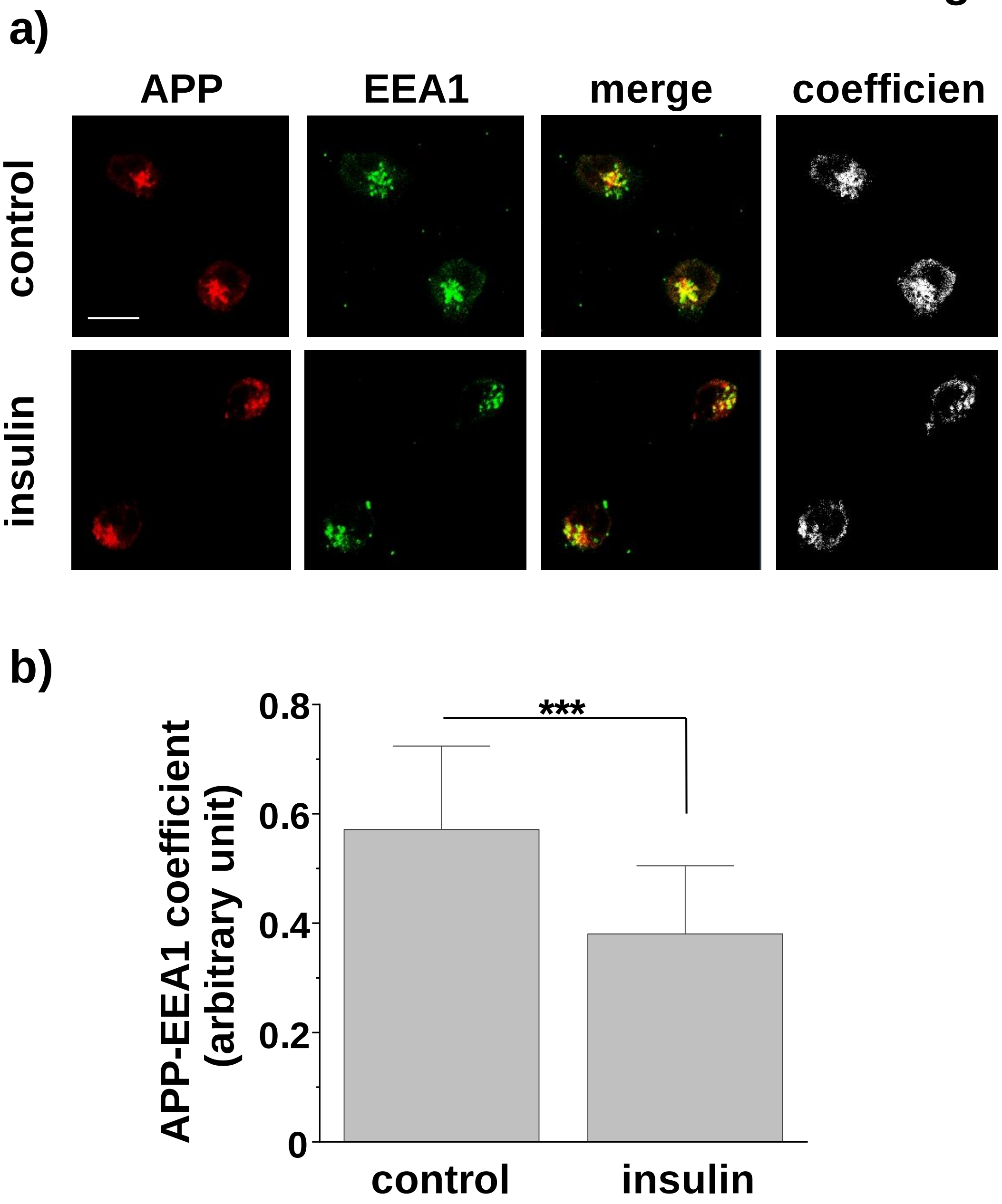
a)
APP
EEA1
merge
coefficien
control
insulin
b)
APP-EEA1 coefficient
(arbitrary unit)
0.8
0.6
0.4
0.2
0
***
control
insulin

Figure S13. Insulin decreased the localization of internalized APP in early endosomes. SH-SY5Y-APP/BACE1 cells were treated with the 6E10 antibody at 4°C for labeling of APP in plasma membrane. After washing, cells were incubated at 37°C for 5 min to allow endocytosis. Then, cells were fixed, permeabilized, and incubated with EEA1 (marker for early endosome antibody). (a) Representative confocal image shows the co-localization of internalized APP (red) and EEA1 (green) at 5 min. (b) The coefficient of internalized APP and EEA1 was significantly decreased in insulin-treated cells compared to control cells. The intensities were quantified as the arbitrary unit by LSM710 microscope (Zeiss) using Zen software (n=20, number of cells. Randomly sectected cells in 2 different experiments). The threshold was automatically set as the 60-100% value. (Manders' M1 coefficient: control, 0.56629±0.02014; insulin, 0.37574±0.01645; fraction of B overlapping A, image A: APP, image B: EEA1). Scale bar is 10 μm. One way ANOVA: ***, $p < 0.001$. All values represent mean ± SEM.
